# Discrete Heterotrimetallic Assemblies Based on Rod‐Shaped Fe^II^‐Metalloligands and a Zn^II^‐Porphyrin/Ru^II^‐Metallacycle

**DOI:** 10.1002/chem.202501811

**Published:** 2025-07-17

**Authors:** Agnese Amati, Giacomo Cecot, Irene Regeni, Erica Giraldi, Kay Severin, Nicola Demitri, Elisabetta Iengo

**Affiliations:** ^1^ Department of Chemical and Pharmaceutical Sciences University of Trieste Via L. Giorgieri 1 Trieste 34127 Italy; ^2^ Institut des Sciences et Ingénierie Chimiques École Polytechnique Fédérale de Lausanne (EPFL) Lausanne 1015 Switzerland; ^3^ Institut de Science et d'Ingénierie Supramoléculaires University of Strasbourg 8 allée Gaspard Monge, BP 70028 Strasbourg F‐67083 Cedex France; ^4^ Elettra–Synchrotron Light Source S.S. 14 Km 163.5 in Area Science Park, 34149 Basovizza Trieste Italy

**Keywords:** assemblies, Fe^II^‐metalloligand, heterometallic, Ru^II^‐metallacycle, Zn^II^‐porphyrin

## Abstract

An efficient strategy for the preparation of heterometallic discrete porphyrin assemblies, tuned both in dimensions and number of metal centers, is described. Five rod‐shaped di‐pyridyl Fe^II^‐metalloligands, with varied length (1.5 – 3.2 nm), lateral substituents, and number of iron centers, were used to bridge two Ru^II^‐metallacycles, made of two coplanar Zn^II^‐porphyrin each. The resulting architectures consist of four Ru^II^ complexes, four zinc‐porphyrins, and either two or four Fe^II^‐clathrochelate units. Earlier, geometrically similar *sandwich‐like* architectures were based on purely organic connectors. Among other novel characteristics, the use of metalloligands was found to be beneficial for the overall stability, thus allowing for a solution‐based characterization of the assemblies. Single crystal X‐ray structures were determined for the complete collection, highlighting additional key features: the two facing Zn^II^‐porphyrin platforms are set wide apart according to the span of the two connecting metalloligands, while the latter are parallelly aligned by the anchoring Zn^II^‐porphyrin/Ru^II^‐metallacycles, at fixed inter Fe··Fe distance(s). Mutual control over these geometrical parameters is very strict, as evidenced by self‐sorting experiments. Useful implementation of these systems into functional systems may be envisaged by pairing the peripheral metalloporphyrin photosensitizers with photo/redox/catalytically active inner metal cores.

## Graphical abstract

Modular self‐assembly of extendable porphyrin‐based heterotrimetallic discrete architectures: a new efficient strategy toward the construction of stable multicomponent functional systems.

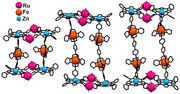

## 1 Introduction

**Figure 1:**
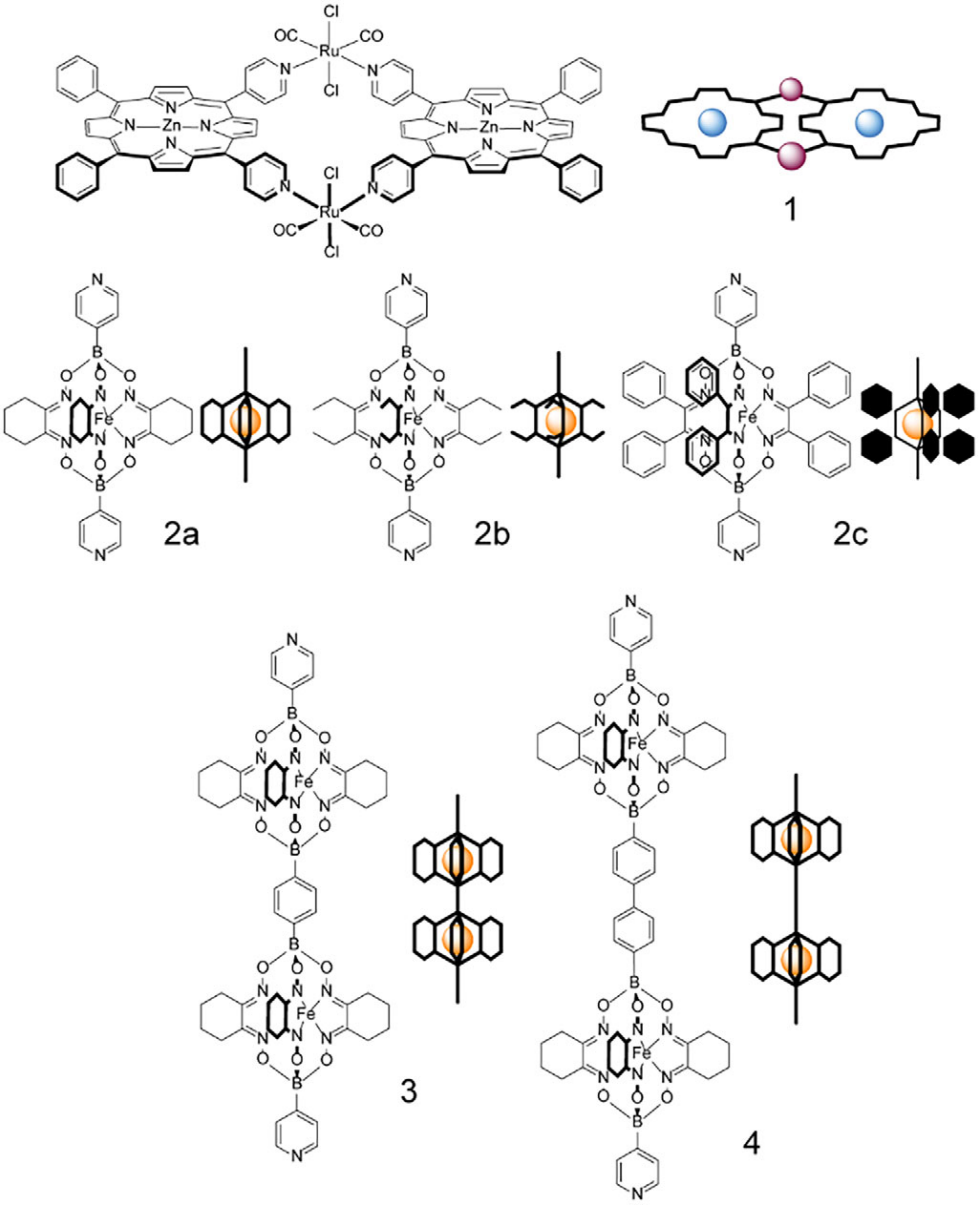
Structures and schematic representations of the zinc‐porphyrin metallacycle **1**, and of the rod‐shaped dipyridyl metalloligands **2**–**4**.

From nature's intricate machinery to cutting‐edge technology, (metallo)porphyrins serve as versatile molecular synthons. Their remarkable photophysical properties, exploited by nature in essential processes such as photosynthesis and oxygen transport, make them ideal for applications ranging from molecular recognition to catalysis. Among other synthetic strategies, the metal‐mediated approach has allowed the synthesis of a plethora of multiporphyrinic discrete structures, with emerging properties, in high yields;^[^ ^1^, ^2^ ^]^ these usually contain *meso*‐substituted porphyrins, and are most often homometallic, while there is currently a strong interest in the design of functional multicomponent metal‐based systems.^[^ ^3^, ^4^, ^5^ ^]^ Typical approaches are based on chemoselective self‐sorting via either hard/soft discriminations, coordination geometry preferences, or kinetic control.^[^ ^6^, ^7^, ^8^, ^9^, ^10^, ^11^, ^12^, ^13^, ^14^ ^]^ The Iengo group introduced a modular strategy for the assembly of multiporphyrin 3D discrete systems, built on the Ru^II^‐metallacycle of Zn^II^‐porphyrins [*t,c,c*‐RuCl_2_(CO)_2_(Zn⋅4′cisDPyP)]_2_ (**1**) (Figure 1).^[^ ^15^, ^16^ ^]^ Complex **1** can be obtained by reaction of *t,c,c*‐RuCl_2_(CO)_2_(dmso‐O)_2_ with 4′‐cis‐dipyridylphenylporphyrin (4′cisDPyP), followed by metalation with Zn(OAc)_2_. The metallacycle adopts a flat geometry with two coplanar porphyrinth units, both in solution and in the solid state.^[^ ^15^, ^16^ ^]^ While the two Ru complexes are electronically saturated and kinetically inert, the zinc(II) centers are Lewis acidic and can bind to N‐donor ligands. Thus, **1** can be conveniently described as a platform with two terminal acceptor sites. The combination of **1** with different polytopic organic nitrogen ligands, in an appropriate ratio, permitted the efficiently and quantitatively assemble a variety of multiporphyrin 3D discrete architectures.^[^ ^15^, ^17^, ^18^, ^19^, ^20^, ^21^ ^]^ Noteworthy, for the *sandwich‐like* assemblies, bearing two peripheral platforms and two linear photoactive connectors, the appealing emergence of inter‐component photoinduced processes was observed, differing in dependence on the nature of the connectors.^[^ ^20^, ^21^ ^]^ We now report on the use of linear metalloligands, in place of organic connectors, to synthetizes a library of sandwich‐like heterometallic Zn^II^‐porphyrins/Ru^II^ derivatives. In general, the implementation of metalloligands –, that is, metal complexes with available donor groups at their periphery – can bestow the final supramolecular assembly with interesting new functions and properties such as catalytic or redox activity, and magnetism.^[^ ^22^, ^23^, ^24^, ^25^, ^26^, ^27^, ^28^, ^29^, ^30^ ^]^ The Severin group has previously shown that boron‐capped Fe^II^‐clathrochelate complexes with terminal donor groups are versatile building blocks for the construction of various multimetallic 3D cages.^[^ ^22^, ^31^, ^32^, ^33^, ^34^, ^35^, ^36^, ^37^ ^]^ The synthetic approach for the preparation of these metalloligands is highly efficient and conveniently modular. This allows for a free choice over the number/type/position of the apical donor groups, the substitution pattern, the overall length of the ligand, the number of clathrochelate cores, and the nature of the metal(s).^[^ ^30^, ^31^, ^32^, ^33^, ^34^, ^35^, ^36^, ^37^, ^38^, ^39^, ^40^ ^]^ For instance, metalloligands **2a**–**c**, bearing different lateral substituents (Figure 1), can be prepared by one‐pot reactions of FeCl_2_, 4‐pyridylboronic acid, and the dioxime with the desired groups (i.e., –R = ethyl, cyclohexyl, di‐phenyl, respectively). The extended metalloligands **3** and **4**, featuring two clathrochelate cores (Figure 1), are in place isolated by selective precipitation after reaction of the appropriate mixture of a mono and a diboronic acid, the chosen dioxime, and FeCl_2_. It is noteworthy that ligand **4** has a length of around 3.2 nm. While ditopic 3‐pyridyl or multi‐topic 4‐pyridyl metalloligands were used extensively for the successful construction of an ensemble of metallacages,^[^ ^41^, ^42^, ^43^, ^44^ ^]^ the exploitation of the dipyridyl rod‐shaped version was more limited.^[^ ^45^, ^46^, ^47^, ^48^, ^49^, ^50^ ^]^ So far, it has been restricted to the preparation of Cr_7_Ni‐ring nanomagnets,^[^ ^47^ ^]^ the assembling of either Re^I^‐ or Ir^III^‐based hetero‐metallacycles,^[^ ^48^, ^49^ ^]^ and infinite heterometallic MOF architectures.^[^ ^50^ ^]^

**Figure 2:**
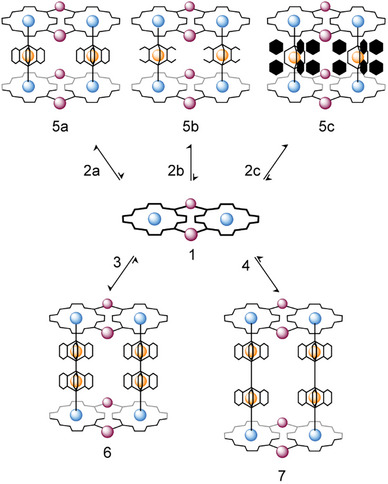
Schematic representations of the 1:1 assembling processes between **1** and each of the metalloligands **2**–**4** (CHCl_3_, R.T.), with instant formation, in nearly quantitative yields, of the discrete heterometallic assemblies **5**–**7**.

Concerning the clathrochelate lateral substitution pattern, it was previously observed that the use of ligands with different side chains or length‐to‐width ratios can be pivotal factors for the self‐assembly outcomes.^[^ ^34^, ^35^, ^51^ ^]^ Below, we show that the combination of platform **1** with linear dipyridyl metalloligands **2a**–**c**, **3**, or **4** (Figure 1) affords, in quantitative yields, large heteroleptic discrete assemblies of general formula {[*t*,*c*,*c*‐RuCl_2_(CO)_2_(Zn·4′cisDPyP)]_2_}_2_{4′‐dipyridyl‐Fe_n_^II^(clathrochelate)}_2_ (*n* = 1: **5a**–**c**, *n* = 2: **6**, **7**, Figure 2). The rigidity and the variable lengths of the rod‐shaped Fe^II^‐connectors govern with high precision the distance of the two Zn^II^‐porphyrin metallacycles, and thus the extension of the derivatives; the Zn^II^ Lewis acid centers of **1** act as scaffolds and accurately pinpoint the location of two or twosome iron cores (in **5a**–**c** or in **6**, **7**, respectively); the lateral inner space is large enough to accommodate pairs of identical metalloligands with varied side hindrance. Moreover, the inertness of the Ru − pyridyl bonds of **1** and of the clathrochelate iron cores of **2**–**4** is crucial in preventing ligand or metal scrambling reactions during the assembling process. All the derivatives are formed following an *all‐or‐nothing* process; the increased stability of **5**–**7**, with respect to the previously reported sandwiches, brings these systems in the slow exchange regime on the NMR timescale, already at room temperature. This unprecedented feature allowed us to perform a detailed solution‐based characterization, with new useful insights (e.g., DOSY analysis, identification of dynamic exchange processes, and self‐sorting experiments). Moreover, the high degree of control over the purity of the samples was essential for the isolation of single crystals and successful X‐ray data analyses for the whole set of assemblies. The future perspective may be that of translating this reliable modular approach to more appealing, functional supramolecular systems, featuring defined and spatially organized numbers of metal‐active cores and photosensitizer units.

## 2 Results and Discussion

For the present study, we have investigated reactions of platform **1** with the rod‐shaped Fe^II^‐metalloligands **2a**–**c**, **3,** and **4** (Figure 1). This systematic survey appeared interesting in order to verify: i) the efficacy of the previously established assembling approach in the presence of rigid (and also) substantially more extended connectors; ii) the degree of mutual control, exerted by the preorganization of **1** and **2**–**4**, over the spatial distribution of the various metal centers and peripheral chromophores; iii) the possibility to self‐sort mixed metalloligand assemblies.

**Figure 3:**
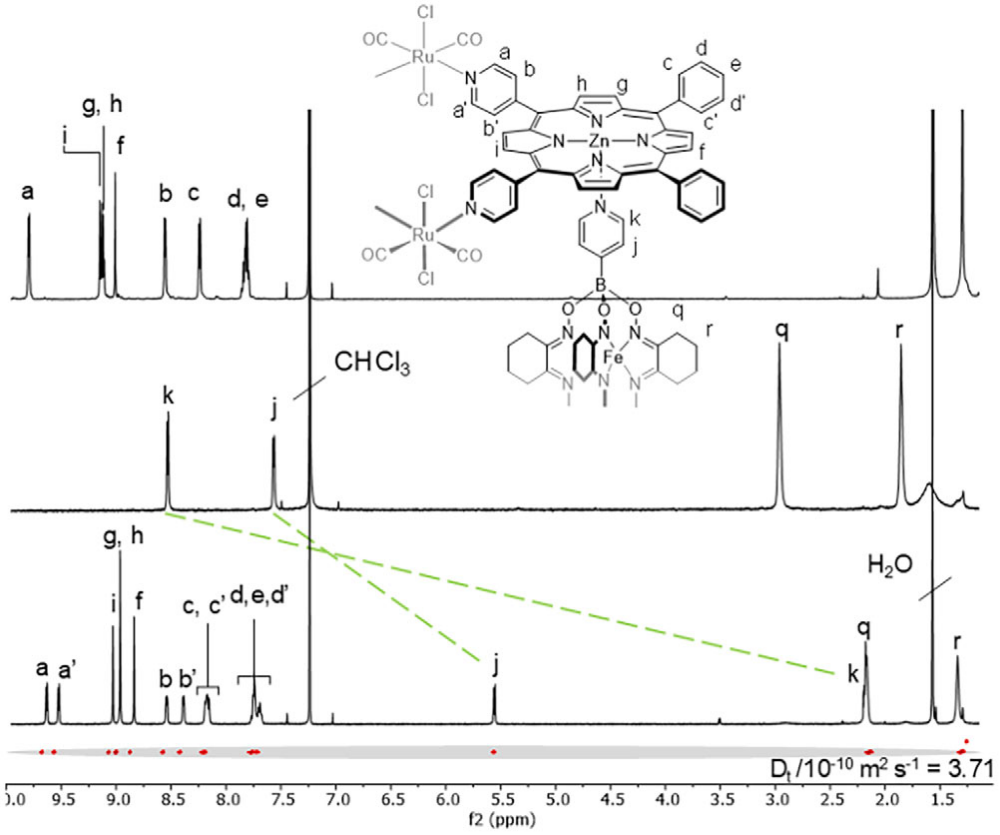
^1^H NMR spectra (CDCl_3_) of **1** (top), **2a** (middle), and **5a** (bottom), with proton labeling scheme; 2D ^1^H DOSY trace of **5a**. Due to the reduced symmetry of **5a**, and slow rotation of the porphyrin meso substituents, splitting into resonances of equal intensities are observed for protons H_a–d_.

Treatment of **1** in chloroform solution at room temperature with one eq. of ligands **2a**–**c** led to the quantitative formation of systems **5a**–**c** (Figure 2), isolated as purple microcrystalline solids by precipitation with *n*‐hexane. The three heterotrimetallic adducts present common features consistent with the unique formation of a discrete *sandwich‐like* architecture of formula {[*t,c,c*‐RuCl_2_(Zn·4′cisDPyP]_2_}_2_{4′‐dipyridyl‐Fe^II^(clathrochelate)}_2_. A complete and unambiguous characterization of **5a**‐**c** was done in solution by means of in‐depth NMR analysis, IR, absorption, and emission spectroscopies (see also SI). A concise description of the corresponding spectroscopic “fingerprints” is given for **5a** (Figure 3). At room temperature, the ^1^H NMR spectrum of **5a** presents one set of sharp and well‐resolved resonances, with the correct relative integration, highlighting marked kinetic inertness and thermodynamic stability. Further ^1^H NMR experiments at variable temperature (ΔT = +45 ÷ −45 °C) did not show appreciable variations, indicating that the assembly maintains its stability and inertness in a wide temperature range (Figures S19/20). These observations represent the most striking difference with the previously reported systems,^[^ ^15^, ^21^ ^]^ and permitted to gather new insights through an in‐depth solution study.

The coordination of the terminal 4‐pyridyl groups of **2a** to the Zn‐porphyrin units of **1** is indicated by the characteristic high field shifts of the corresponding proton resonances (e.g., Δ*δ* = −6.42 ppm for *H*_k_, Figure 3).^[^ ^15^, ^21^ ^]^ The ^1^H NMR signals belonging to the pyridyl and *ortho* plus *meta* phenyl groups of platform **1**, on the other hand, are found to be split into two sets of resonances of equal intensity, in line with the reduced symmetry of complex **5a** compared to free **1**, and with the slow rotation on the NMR time scale of the porphyrin aryl substituents (Figure 3).^[^ ^52^ ^]^ Clear exchange peaks between the pairwise split signals can be observed in the ^1^H‐^1^H ROESY spectrum; in the same 2D spectrum spatial, correlations allow for the univocal assignment of all the β‐pyrrolic porphyrin resonances (Figure S13–15).

Despite the difference in flexibility and steric demand of the side chains of **2a**–**2c**, all the resulting assemblies were found to be highly symmetric, thus implying a substantial degree of rotational freedom of the two rod‐shaped connectors around the longitudinal axes, independently from the ligand width (in solution, on the NMR time scale, over the T range explored). Quite expectedly, the mass spectroscopic analyses were unsuccessful,^[^ ^53^, ^54^ ^]^ therefore Diffusion‐Ordered Spectroscopy (DOSY) was used to recover additional information on their size. 2D DOSY experiments of systems **5a**–**c** show only one species to be present in solution (Figure 3 and Figures S24–28), in line with the formation of the described adducts (see also below).

Having established that the metalloligands **2a**–**c** and platform **1** can be used for the construction of stable heterometallic constructs, we next investigated reactions with the more extended bis‐clathrochelates **3** and **4** (featuring the same cyclohexyl substituents, but different Fe^II^‐Fe^II^ spacers). First, we performed in situ NMR spectroscopic analyses of mixtures containing either **3** or **4** and equimolar amounts of platform **1**. The NMR data provided clear evidence for the quantitative and immediate formation of the discrete *sandwich‐like* supramolecular assemblies **6** and **7**, formulated as {[*t,c,c*‐RuCl_2_(Zn·4′cisDPyP]_2_}_2_{4′‐dipyridyl‐Fe_2_^II^(clathrochelate)}_2_ (Figure 2, and SI). As in the case of **5a**–**c**, we observed strong‐high field shifts for the protons of the metalloligands (progressively less marked as the distance from the shielding porphyrins increases, along with a splitting of the signals from the aryl *ortho* and *meta* protons of **1** (Figures S16/S17). Therefore, the modular self‐assembling approach remains quantitative also when the connectors span a considerable length between the two basic pyridyl donor sites (no evidence of oligomeric side products). In Table S1, the values of the diffusion coefficient, hydrodynamic radius, and volume, calculated from the Stokes‐Einstein equation, for the whole series together with the starting building blocks, are reported. These values give information about the size of the species, while the most interesting indications can be inferred by comparison of those values. The trend observed for both *D*_t_ and *r*_H_ of **5a**, **6,** and **7**, in which the longitudinal ligand length considerably increases, is consistent with the formation of progressively more extended architectures. Interestingly, metalloligands **2a**–**c** of the same length but distinct lateral width, are size‐discriminated by DOSY analysis only when free, while their corresponding assemblies **5a**–**c** afford similar *D_t_* and *r*_H_ values. These observations indicate that the side space spanned by platform **1** is equally inclusive for the whole metalloligands set. The remarked kinetic inertness of **5**–**7** also allows to unambiguously establish that the assembly process between **1** and each of the metalloligands occurs instantly via an *all‐or‐nothing*reversible process.^[^ ^55^ ^]^ When an excess of **1** was added to a CDCl_3_ solution of whichever metalloligand, the ^1^H NMR spectrum, recorded immediately after the addition, showed only two sets of sharp signals: one corresponding to the fully formed assembly, and the other to the residual excess **1** (Figure S18), with related exchange correlations in the ^1^H‐^1^H ROESY spectrum at room temperature. The enhanced stability of **5**–**7** may be in part explained by considering the higher basicity of the pyridyl groups of metalloligands **2**–**4**, compared to that of the donor groups in the original purely organic connectors,^[^ ^15^, ^21^ ^]^ and likely related to the presence of a boronate ester group with a formal negative charge.^[^ ^56^, ^57^, ^58^, ^59^, ^60^, ^61^, ^62^ ^]^ Qualitative spectrofluorimetric experiments were carried out in order to monitor the solution stability of the novel assemblies. By following the emission maxima blue shift upon dilution, concomitant with dissociation of the N‐ligands from the Zn^II^‐porphyrin platforms, the lower limit concentration at which **5**–**7** are still intact was found to be ca. 2 × 10^−5^ M (Figures S34/S35).

**Figure 4:**
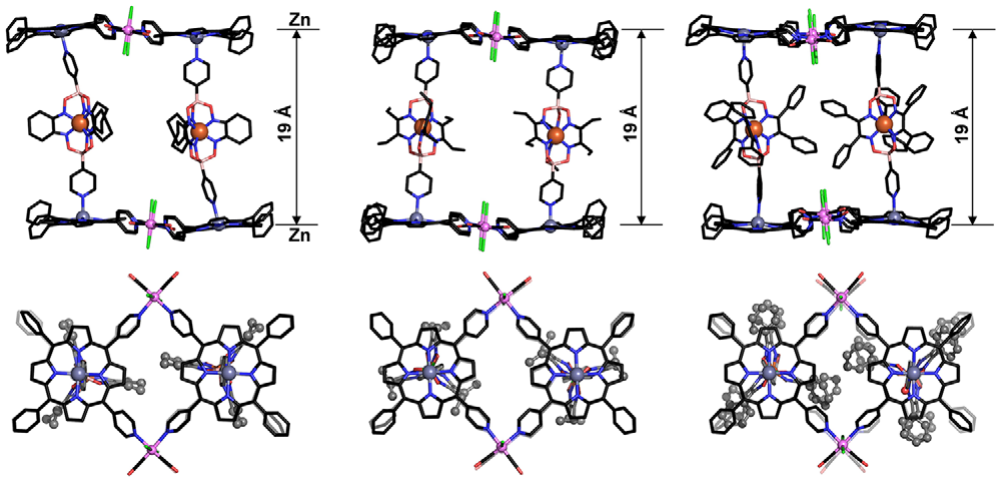
Two views of the molecular structures of **5a**–**c**, with longitudinal dimensions; solvent molecules and hydrogen atoms are omitted for clarity. Color coding: black sticks for C, blue for N, red for O, pink for B, violet for Ru, orange for Fe, purple for Zn, green for Cl.

**Figure 5:**
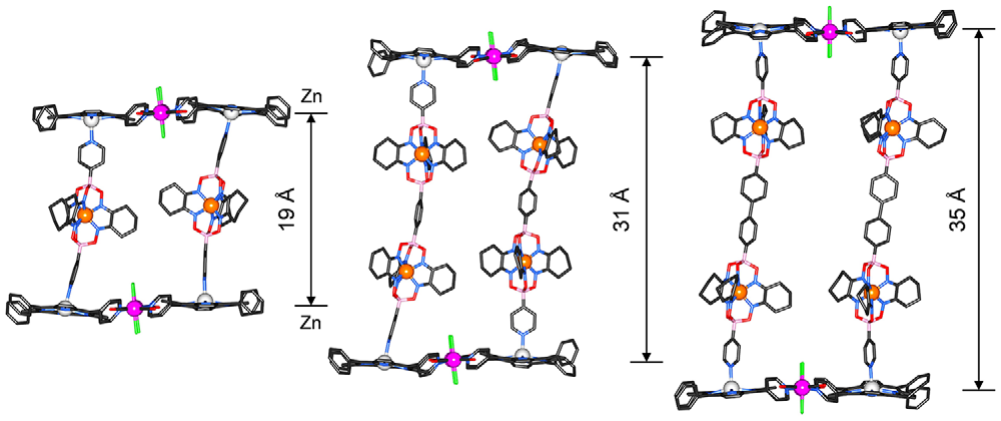
Molecular structures of **5a**, **6**, and **7**, with longitudinal dimensions; solvent molecules and hydrogen atoms are omitted for clarity. Color coding: black sticks for C, blue for N, red for O, pink for B, violet for Ru, orange for Fe, purple for Zn, green for Cl.

Single crystals of **5a**–**c**, **6,** and **7** were obtained by slow diffusion of *n*‐hexane into chloroform solutions of each assembly, and X‐ray analyses revealed for all of them the expected *sandwich‐like* structures, consisting of two Zn‐porphyrin metallacycles connected *face*‐to‐*face* by two bridging metalloligands (Figures 4, 5).

The most relevant structural parameters of the assemblies are summarized in Table 1. All derivatives possess an inversion center at the barycenter, such that only half of each assembly occupies the corresponding crystallographic asymmetric units (ASU, Figure S37). This arrangement leads to a perfectly parallel alignment of the two zinc‐porphyrin platforms. Consistent with the trends observed in the DOSY data (Table S1), **5a**–**c** exhibit similar Zn···Zn distances of approximately 19 Å (Table 1 and Figure 4, top). On the other hand, and in line with the pyridine···pyridine increased distances of ligands **3** and **4**, assemblies **6** and **7** present considerable longitudinal dimensions of 31 Å and 35 Å (Table 1 and Figure 5). As shown in the side and top views of **5a**–**c** in Figure 4, the side chains of ligands **2a**–**c** are characterized by different spatial arrangements. In **5a** and **5b**, the two bridging ligands are relatively far from one another, with no apparent steric clashes or contacts. In contrast, in assembly **5c,** a gear‐like arrangement of the metalloligands aromatic groups is observed, and the central phenyl rings are found to interact via π‐π stacking (shortest C⋅⋅⋅C distance of 3.68 Å, Figure S45). For **5a**, two different triclinic crystal forms were produced under similar crystallization conditions (identified as **5a** and **5a′**, Table 1). These forms differ in the conformations of the bridging ligands within the sandwich structure (see also Figures S37/38). Additional details, pictures, and crystallographic data are given in the Supporting Information.^[^ ^63^, ^64^ ^]^

**Table 1:** Selected distances [Å] for **5a**–**c**, **6**, and **7**.

	Zn•••Zn_long_ ^[a]^	Zn•••Zn_short_ ^[b]^	Fe•••Fe ^[c]^	N•••N^[d]^	Ru•••Ru^[e]^
**5a**	18.950(5)	13.899(8)	12.572(7)	14.74(1)	13.924(4)
**5a′**	18.838(8)	13.568(7)	11.932(8)	14.67(3) [14.950(4)]^[g]^	14.079(6)
**5b**	19.026(5)	13.756(4)	13.683(4)	14.75(2)	14.131(4)
**5c**	18.92(1)	13.714(3)	13.063(3)	14.700(8)	14.050(5)
**6**	31.05(1)	14.072(4)	12.062(4) [12.091(4)]^[g]^ [11.204(2)]^[h]^	26.80(2) [26.82(1)]^[g]^	13.885(5)
**7**	35.24(1)	13.811(4)	13.141(7) [13.070(4)]^[h]^	31.00(2)	14.103(6)
**8** ^[f]^	19.463(7)	13.532(5)	[11.455(2)]^[h]^	15.18(2)	14.454(5)

^[a]^ Distance between two Zn atoms connected by a single metalloligand.

^[b]^ Distance between two Zn atoms of the same Zn^II^‐porphyrin platform.

^[c]^ Distance between two aligned Fe atoms of two parallel metalloligands

^[d]^ Distance between terminal N atoms of a single metalloligand.

^[e]^ Distance between two Ru atoms of the same Zn^II^‐porphyrin platform.

^[f]^ Values calculated from the reported structure of the sandwich reference assembly {[*t,c,c*‐RuCl_2_(Zn·4′cisDPyP]_2_}_2_{4′transDPyP}_2_ (**8**, Figure S3).^[^ ^15^ ^]^

^[g]^ Values calculated from the reported structures of the isolated ligands.^[^ ^49^ ^]^

^[h]^ Distance between the centroids of the aligned phenyl spacer(s) in **6** or **7**, or between the centroids of the two aligned 4′transDPyP porphyrins in **8**.

**Figure 6:**
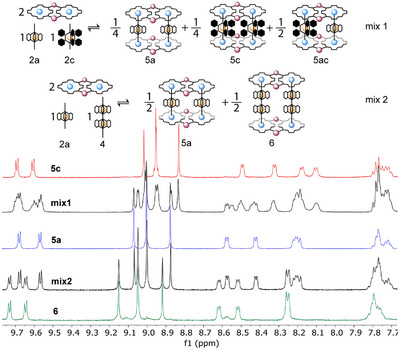
Top: schematic depiction of the equilibrium mixtures forming by combination, in a 2:1:1 ratio (ca. 10^−3^ M; CDCl_3_; room temperature) of: **1**, **2a**, and **2c** (**mix1**), as well as **1**, **2a**, and **3** (**mix2**). Bottom: overlay of the ^1^H NMR spectra (aromatic region, CDCl_3_) of **5c** (red), **mix1** (black), **5a** (blue), **mix2** (black), and **6** (green).

We then investigated the self‐sorting behavior of ligands **2a**, **2c**, and **3**, in the presence of platform **1**, to elucidate the possible effect of the longitudinal and lateral dimensions of the Fe^II^‐ligand connectors. We started by mixing **2a**, **2c** and **1** in a 1:1:2 ratio (mix1, Figure 6). Both ligands of this mixture are characterized by the same length but differ in their width. The ^1^H NMR spectrum of **mix1** evidenced the statistical formation of the two homoligand systems **5a** and **5c**, and the heteroligand one (indicated as **5ac** in Figure 6). Formation of a mixture in strict statistical fashion, as depicted in Figure 6, is unlikely, but a precise distribution analysis of **mix1**, as well as direct proof of the composition of **5ac**,^[^ ^65^ ^]^ could not be inferred due to extended overlap of the ^1^H NMR signals. Still, the comparison of the ^1^H NMR spectra in Figure 6 is quite convincing, and the behavior observed is in contrast with the observations on previous reported examples by Severin. For those systems, the occurrence of steric clashes between side groups of adjacent connectors turned advantageous for the self‐sorting of mixed assemblies over homoleptic ones, or alternatively, favorable interactions between bulky side groups of nearby ligands drove the formation of a specific coordination cage,^[^ ^34^, ^35^, ^51^ ^]^ no efficient self‐sorting was observed in our case (while favorable π−π stacking between interdigitating aromatic substituents of the metalloligands in **5c** was observed in the solid state, see above). On the other hand, by mixing ligands **2a** and **3** with platform **1** in a 1:1:2 ratio, only the peaks relative to the homoligand assemblies **5a** and **6** are present, corresponding to a narcissistic self‐sorting behavior (**mix2**, Figure 6). The same results were obtained by mixing in a 1:1 ratio the preformed homoligand assemblies **5a** and **5c**, or **5a** and **6**, respectively. Not surprisingly, the ^1^H‐^1^H ROESY experiments of both mixtures show clear exchange peaks between the zinc‐porphyrin platforms pertaining to distinct assemblies in the mixtures (see also Figures S29–33).

## 3 Conclusion

The present study demonstrates the robustness and versatility of the modular self‐assembly approach to generate large, discrete, and structurally defined heterometallic discrete architectures through the integration of a Zn^II^‐porphyrin/Ru^II^‐metallacycle and Fe^II^ clathrochelate‐based metalloligands. The precise spatial organization and exceptional stability of these sandwich‐like assemblies—enabled by the preorganized design of both platform and connectors—open new avenues for detailed structural and spectroscopic investigations in solution and solid state. Beyond their structural elegance, these systems highlight the potential of metalloligands to impart additional functionality. Looking forward, this synthetic strategy may be extended to even more complex heterometallic constructs, where deliberate placement of diverse redox‐ and spin‐active centers could enable systematic exploration of cooperative or competing magnetic interactions, laying the groundwork for multifunctional supramolecular magnetic materials.

## Supporting Information

The authors have cited additional references within the Supporting Information.^[^ ^66^, ^67^, ^68^, ^69^, ^70^, ^71^, ^72^, ^73^, ^74^, ^75^, ^76^, ^77^, ^78^, ^79^, ^80^ ^]^ Experimental Section and complete solution characterization, together with additional X‐ray data and pictures,^[^ ^63^ ^]^ are available in the supporting information of this article.

## Conflict of Interest

The authors declare no conflict of interest.

## Supporting information



Supporting Information

## Data Availability

The data that support the findings of this study are available in the supplementary material of this article.
