# Supplementary material for: Discrete Heterotrimetallic Assemblies Based on Rod‐Shaped FeII‐Metalloligands and a ZnII‐Porphyrin/RuII‐Metallacycle
Source: Chemistry. 2025 Jul 17;31(44):e202501811. doi: 10.1002/chem.202501811 (PMC12336794; doi:10.1002/chem.202501811)
Supplement: Supplementary file 1 — Supporting Information [file CHEM-31-e202501811-s001.pdf]

SUPPORTING INFORMATION

Discrete Heterotrimetallic Assemblies Based on Rod-Shaped Fe^{II}-Metalloligands and a Zn^{II}-Porphyrin/Ru^{II}-Metallacycle

Agnese Amati,[a] Giacomo Cecot,[b] Irene Regeni,[a,c] Erica Giraldi,[a,b] Kay Severin,[b] Nicola Demitri,*[d] and Elisabetta Iengo*[a]

[a] Dr. A. Amati, Dr. I. Regeni, Dr. E. Giraldi, Prof. Dr. E. Iengo*
Department of Chemical and Pharmaceutical Sciences
University of Trieste
Via L. Giorgieri 1, 34127 Trieste, Italy
E-mail: eiengo@units.it
[b] Dr. G. Cecot, Dr. E. Giraldi, Prof. K. Severin
Institut des Sciences et Ingénierie Chimiques
École Polytechnique Fédérale de Lausanne (EPFL)
1015 Lausanne, Switzerland
[c] Current Address:
Institut de Science et d'Ingénierie Supramoléculaires
University of Strasbourg
8 allée Gaspard Monge, BP 70028, F-67083 Strasbourg Cedex, France
[d] Dr. N. Demitri*
Elettra – Synchrotron Light Source
S.S. 14 Km 163.5 in Area Science Park, 34149 Basovizza, Trieste, Italy
E-mail: nicola.demitri@elettra.eu

Experimental Section

Instrumentations and experimental procedures

NMR. 1D and 2D NMR (1D ^{1}H,^{13}C, ^{1}H DOSY and 2D ^{1}H-^{1}H COSY, ^{1}H-^{13}C HSQC, ^{1}H-^{1}H ROESY) experiments were recorded on a Varian Innova 500 (operating at 500 MHz for ^{1}H and 125 MHz for ^{13}C). All spectra were recorded at 298 K and the ^{1}H chemical shifts were referenced to the peak of residual non-deuterated solvents for ^{1}H: $CDCl_3$, δ = 7.26 ppm and for ^{13}C: $CDCl_3$, δ = 77.16 ppm. For 2D ^{1}H DOSY the *Bipolar Pulse Pair Stimulated Echo* with *convection compensation sequence* (bppste_cc) of VnmrJ software was implemented on the Varian spectrometer. For the DOSY analysis, the Bayesian DOSY transform from MestreNova was employed.

ESI-MS analysis. Mass spectra were recorded on a ThermoScientific Orbitrap Exploris 240 by Dr. Fabio Hollan of the University of Trieste (Italy).

IR Spectroscopy. Infrared spectra were recorded on a Perkin Elmer Spectrum One Golden Gate FT/IR spectrometer in the transmission mode.

UV-Vis Absorption Spectroscopy. Absorption spectra were recorded in 1 cm path length quartz cell with an Agilent Cary 60 spectrophotometer.

Emission Spectroscopy. Emission spectra were recorded at 25 °C in 1 cm path length quartz cell with a Varian Cary Eclipse Fluorescence Spectrophotometer equipped with a Varian Cary Single Cell Peltier **SPVF-1X0** temperature controller.

X-ray Analysis. 2aTPP$_2$ structure has been obtained from data collected on a conventional Mo-Kα source (Bruker APEX II CCD), processed through Bruker AXS Software suite. Data collections on the other crystals were performed at the X-ray diffraction beamline (XRD1) of the Elettra Synchrotron, Trieste (Italy).[66] The crystals were dipped in NHV oil (Jena Bioscience, Jena, Germany) and mounted on the goniometer head with a nylon loop. Complete datasets were collected at 100 K (nitrogen stream supplied through an Oxford Cryostream 700 - Oxford Cryosystems Ltd., Oxford, United Kingdom) through the rotating crystal method. Data were acquired using a monochromatic wavelength of 0.700 Å, on a Pilatus 2M hybrid-pixel area detector (DECTRIS Ltd., Baden-Daettwil, Switzerland). The diffraction data were indexed and integrated using XDS.[67] Scaling has been done using CCP4-Aimless code.[68,69] Complete datasets for all the triclinic crystal forms of the molecules of interest have been obtained by merging data collected from two different random orientations of one or two crystals. The structures were solved by the dual space algorithm implemented in the SHELXT code.[70] Fourier

analysis and refinement were performed by the full-matrix least-squares methods based on F^2 implemented in SHELXL (Version 2017/1).[70] The Coot program was used for modeling.[71] Anisotropic thermal motion refinement has been used for all atoms with full occupancy. Geometric and thermal motion parameters restraints (DFIX, DANG, SIMU or DELU) have been applied on disordered and poorly defined fragments. Hydrogen atoms were included at calculated positions with isotropic $U_{factors} = 1.2 \cdot U_{eq}$ or $U_{factors} = 1.5 \cdot U_{eq}$ for hydroxyl and methyl groups (U_{eq} being the equivalent isotropic thermal factor of the bonded non hydrogen atom). All the structures contain relevant amount of solvent (in ~40% of cell volume), only partially ordered. Disordered solvent contributions that couldn't be modeled have been removed with Platon SQUEEZE routine[72 (the number of electrons squeezed and the cell volume voids are reported in Table 1S).
Geometrical assembly parameters have been calculated using Diamond software[73] and are reported in Table 1 of the manuscript and in Table S2. Essential crystal and refinement data are reported below in Table S3.
Pictures were prepared using either Ortep3 or Pymol softwares.[74,75]
CCDC 2388760-2388767 contain the supplementary crystallographic data for compounds **2aTPP$_2$**, **2cTPP$_2$**, **5a**, **5a'**, **5b**, **5c**, **6**, and **7**. These data can be obtained free of charge from The Cambridge Crystallographic Data Centre via https://www.ccdc.cam.ac.uk/structures.

Materials and Methods

All reagents were purchased from Sigma-Aldrich and used without further purification, unless otherwise stated. [*t*,*c*,*c*-RuCl$_2$(CO)$_2$(Zn·4′cisDPyP)]$_2$ (**1**), Fe^{II}-metalloligands **5**–**7** and Zn^{II}-*meso*-tetraphenylporphyrin (**Zn·TPP**) were prepared as described earlier.[15,49,76]

Preparation of the assemblies {[*t*,*c*,*c*-RuCl$_2$(CO)$_2$(Zn·4′cisDPyP)]$_2$}$_2${4′-dipyridyl-Fe_n^{II}(clathrochelate)}$_2$ (n = 1: **5a**–**c**, n = 2: **6**, **7**), and of the models {Zn·TPP}$_2${4′-dipyridyl-Fe^{II}(clathrochelate)}$_2$ (**2aTPP$_2$**, **2cTPP$_2$**), followed a common procedure here described for **5a**.

5a: 25.0 mg of **1** (13 µmol) were dissolved in 10 mL of $CHCl_3$ and 8.7 mg of **2a** (13 µmol) were then added. The colour <of the solution turned immediately from purple to deep violet. The homogeneous system was kept under stirring at room temperature for fifteen minutes. Diffusion of *n*-hexane over the deep red solution induced the precipitation of the

pure product as a purple microcrystalline solid that was collected by filtration, washed with *n*-hexane, and vacuum-dried. Yield: 30.1 mg (94%). ^{1}H NMR (500 MHz, $CDCl_3$, 25 °C): δ = 9.68 (d, ^3J(H,H) = 4.9 Hz, 8H; H_a), 9.57 ppm (d, ^3J(H,H) = 4.8 Hz, 8H; $H_{a'}$), 8.98 ppm (m, 32H; H_i, H_g, H_h, H_f), 8.57 ppm (d, ^3J(H,H) = 4.4 Hz, 8H; H_b), 8.42 ppm (d, ^3J(H,H) = 4.7 Hz, 8H; $H_{b'}$), 8.19 ppm (m, 16H; H_c), 7.78 ppm (m, 24H; H_d, H_e), 5.56 ppm (d, ^3J(H,H) = 6.1 Hz, 8H; H_k), 2.14 ppm (s, 32H; H_j, H_q), 1.31 ppm (2, 24H; H_r); ^{13}C NMR (125 MHz, $CDCl_3$, 25 °C, from HSQC): δ = 150.60 (C_a), 150.21 ($C_{a'}$), 142.38 (C_j), 135.58 (C_c), 132.12 (C_b), 132.74 (C_f, C_g, C_h, C_i), 127.34 (C_k), 127.28 (C_d, C_e), 26.02 (C_q), 20.37 (C_r); selected IR bands (KBr): ῦ = 2065, 1999 cm^{-1} (C=O), 1611 cm^{-1} (C=N); UV/Vis (chloroform): λ^{max} (ε mol^{-1}dm^{3}cm^{-1}) = 440 nm (7x10^4), 570 nm, 610 nm.
Red needle-shaped single crystals have been obtained by slow diffusion of *n*-hexane into a chloroform solution of **5a** at room temperature. Two different triclinic crystal forms have been obtained from very similar crystallization conditions, here indicated as **5a** and **5a′**.

5b: The same synthetic procedure described for **5a** was followed starting from 25.0 mg of **1** (13 µmol) and 8.8 mg of **2b** (13 µmol). Yield: 29.7 mg (92%). ^{1}H NMR (500 MHz, $CDCl_3$, 25 °C): δ = 9.69 (d, ^3J(H,H) = 5.1 Hz, 8H; H_a), 9.58 ppm (d, ^3J(H,H) = 5.1 Hz, 8H; $H_{a'}$), 9.09 ppm (s, 8H; H_i), 9.03 ppm (s, 16H; H_g, H_h), 8.89 ppm (s, 8H; H_f), 8.57 ppm (d, ^3J(H,H) = 3.9 Hz, 8H; H_b), 8.48 ppm (d, ^3J(H,H) = 3.9 Hz, 8H; $H_{b'}$), 8.23 ppm (m, 16H; H_c, $H_{c'}$), 7.78 ppm (m, 24H; H_d, H_e, $H_{d'}$), 5.59 ppm (d, ^3J(H,H) = 6.2 Hz, 8H; H_j), 2.22 ppm (d, ^3J(H,H) = 6.2 Hz, 8H; H_k), 2.10 ppm (d, ^3J(H,H) = 7.0 Hz, 24H; H_l), 0.52 ppm (t, ^3J(H,H) = 7.0 Hz, 36H; H_m); ^{13}C NMR (125 MHz, $CDCl_3$, 25 °C, from HSQC): δ = 150.32 (C_a), 149.80 ($C_{a'}$), 141.11 (C_k), 134.61 (C_c, $C_{c'}$), 133.47 (C_g), 132.48 (C_f), 132.34 ($C_{b'}$), 132.14 (C_b), 130.90 (C_i), 130.86 (C_h), 127.59 (C_e), 126.55 (C_d), 126.25 ($C_{d'}$), 125.14 (C_j), 19.19 (C_l), 10.74 (C_m); selected IR bands (KBr): ῦ = 2065, 1999 cm^{-1} (C=O), 1611 cm^{-1} (C=N); UV/Vis (chloroform): λ^{max} (ε mol^{-1}dm^{3}cm^{-1}) = 441 nm (7x10^4), 570 nm, 611 nm.
Red needle-shaped single crystals have been obtained by slow diffusion of *n*-hexane into a chloroform solution of **5b** at room temperature.

5c: The same synthetic procedure described for **5a** was followed starting from 25.0 mg of **1** (13 µmol) and 12.6 mg of **2c** (13 µmol). Yield: 33.2 mg (92%). ^{1}H NMR (500 MHz, $CDCl_3$, 25 °C): δ = 9.70 ppm (d, ^3J(H,H) = 5.4 Hz, 8H; H_a), 9.61 (d, ^3J(H,H) = 5.4 Hz, 8H; $H_{a'}$), 9.01 ppm (s, 8H; H_i), 8.95 ppm (s, 16H; H_g, H_h), 8.83 ppm (s, 8H; H_f), 8.50 ppm (d, ^3J(H,H) = 3.6 Hz, 8H; H_b), 8.33 ppm (d, ^3J(H,H) = 3.7 Hz, 8H: $H_{b'}$), 8.18 ppm (d, ^3J(H,H) =

6.3 Hz, 8H; H_c), 8.11 ppm (d, ^3J(H,H) = 6.4 Hz, 8H; $H_{c'}$), 7.76 ppm (m, 24H; H_d, H_e, $H_{d'}$), 7.13 ppm (t, ^3J(H,H) = 7.4 Hz, 12H; H_p), 6.86 ppm (t, ^3J(H,H) = 7.7 Hz, 24H; H_o), 6.69 ppm (d, ^3J(H,H) = 7.5 Hz, 24H; H_n), 5.22 ppm (d, ^3J(H,H) = 12.9 Hz, 8H; H_j), 2.03 ppm (d, ^3J(H,H) = 7.0 Hz, 8H; H_k); ^{13}C NMR (125 MHz, $CDCl_3$, 25 °C, from HSQC): δ = 150.26 (C_a), 149.52 ($C_{a'}$), 140.79 (C_k), 134.49 ($C_{c'}$), 134.47 (C_c), 133.33 (C_g), 132.41 (C_f), 132.89 ($C_{b'}$), 131.91 (C_b), 130.79 (C_h), 130.48 (C_i), 129.96 (C_n), 129.71 (C_p), 127.45 (C_e), 127.34 (C_o), 126.49 (C_d), 126.13 ($C_{d'}$), 125.19 (C_j); selected IR bands (KBr): ῦ = 2065, 1999 cm^{-1} (C=O), 1611 cm^{-1} (C=N); UV/Vis (chloroform): λ^{max} (ε $mol^{-1}dm^3cm^{-1}$) = 440 nm (7x10^4), 569 nm, 610 nm.
Red needle-shaped single crystals have been obtained by slow diffusion of *n*-hexane into a chloroform solution of **5c** at room temperature.

6: The same synthetic procedure described for **5a** was followed starting from 25.0 mg of **1** (13 µmol) and 16.3 mg of **3** (13 µmol). Yield: 36.9 mg (93%). ^{1}H NMR (500 MHz, $CDCl_3$, 25 °C): δ = 9.74 ppm (d, ^3J(H,H) = 5.6 Hz, 8H; H_a), 9.66 (d, ^3J(H,H) = 5.6 Hz, 8H; $H_{a'}$), 9.06 ppm (m, 32H; H_f, H_g, H_h, H_i), 8.62 ppm (d, ^3J(H,H) = 5.5 Hz, 8H; H_b), 8.53 ppm (d, ^3J(H,H) = 5.6 Hz, 8H; $H_{b'}$), 8.26 ppm (m, 16H; H_c), 7.79 ppm (m, 24H: H_d, H_e), 7.38 ppm (s, 8H; H_u), 5.72 ppm (d, ^3J(H,H) = 6.0 Hz, 8H; H_k), 2.61 ppm (s, 12H; H_i, H_q), 2.31 ppm (s, 12H; H_t), 2.23 ppm (d, ^3J(H,H) = 6.0 Hz, 8H; H_j), 1.48 ppm (s, 24H; H_r, H_s); ^{13}C NMR (125 MHz, $CDCl_3$, 25 °C, from HSQC): δ = 151.32 (C_a), 150.10 ($C_{a'}$), 142.60 (C_j), 134.24 (C_f, C_g, C_h, C_i), 133.84 (C_c), 133.34 ($C_{b'}$), 132.80 (C_b), 130.80 (C_u), 128.10 (C_k), 127.40 (C_d, C_e), 28.10 (C_q), 27.60 (C_t), 20.80 (C_r, C_s); selected IR bands (KBr): ῦ = 2065, 1999 cm^{-1} (C=O), 1611 cm^{-1} (C=N); UV/Vis (chloroform): λ^{max} (ε $mol^{-1}dm^3cm^{-1}$) = 438 nm (7x10^4), 568 nm, 609 nm.
Red needle-shaped single crystals have been obtained by slow diffusion of *n*-hexane into a chloroform solution of **6** at 4 °C.

7: the same synthetic procedure described for **5a** was followed starting from 25.0 mg of **1** (13 µmol) and 17.3 mg of **7** (13 µmol). Yield: 37.5 mg (92%). ^{1}H NMR (500 MHz, $CDCl_3$, 25 °C): δ = 9.73 ppm (d, ^3J(H,H) = 5.5 Hz, 8H; H_a), 9.66 (d, ^3J(H,H) = 5.7 Hz, 8H; $H_{a'}$), 9.05 ppm (m, 32H; H_f, H_g, H_h, H_i), 8.62 ppm (d, ^3J(H,H) = 6.2 Hz, 8H; H_b), 8.54 ppm (d, ^3J(H,H) = 6.3 Hz, 8H; $H_{b'}$), 8.26 ppm (m, 16H; H_c), 7.80 ppm (m, 24H: H_d, H_e), 7.51 ppm (d, ^3J(H,H) = 9.0 Hz, 8H; H_u), 7.40 ppm (d, ^3J(H,H) = 8.8 Hz, 8H; H_v), 5.74 ppm (d, ^3J(H,H) = 6.0 Hz, 8H; H_k), 2.68 ppm (s, 12H; H_q), 2.34 ppm (s, 12H; H_t), 2.27 ppm (d, ^3J(H,H) = 5.7 Hz, 8H;

H_j), 1.51 ppm (s, 24H, H_r, H_s); ^{13}C NMR (125 MHz, $CDCl_3$, 25 °C, from HSQC): δ = 151.67 (C_a), 151.10 ($C_{a'}$), 143.69 (C_j), 134.70 (C_b), 134.24 (C_f, C_g, C_h, C_i), 133.80 (C_c), 133.67 (C_u), 133.10 ($C_{b'}$), 128.32 (C_d, C_e), 127.29 (C_v, C_k), 27.07 (C_q), 26.13 (C_t), 20.77 (C_r, C_s); selected IR bands (KBr): ῦ = 2065, 1999 cm^{-1} (C=O), 1611 cm^{-1} (C=N); UV/Vis (chloroform): λ^{max} (ε $mol^{-1}dm^3cm^{-1}$) = 438 nm ($7x10^4$), 568 nm, 609 nm.

Red needle-shaped single crystals have been obtained by slow diffusion of *n*-hexane into a chloroform solution of **7** at 4 °C.

2aTPP$_2$: the same synthetic procedure described for **5a** was followed starting from 3.2 mg of **2a** (5 µmol) and 6.6 mg of **ZnTPP** (10 µmol). Yield: 8.9 mg (91%). ^1H NMR (400 MHz, $CDCl_3$, 25 °C): δ = 8.83 (s, 16H; H_f), 8.19 (d, *J*(H,H) = 6.8 Hz, 16H; H_c), 7.69 (m, 24H; H_d, H_e), 5.77 (s, 4H, H_j), 2.84 (s, 4H; H_k), 2.29 (s, 12H; H_q) 1.43 (s, 12H; H_r); ^{13}C NMR (100 MHz, $CDCl_3$, 25 °C, from HSQC): δ = 134.66 (C_c), 131.52 (C_f), 126.45 (C_d, C_e), 125.18 (C_j), 25.72 (C_q), 21.20 (C_r); UV/Vis (chloroform): λ^{max} (ε $mol^{-1}dm^3cm^{-1}$) = 424 nm ($7x10^4$), 553 nm, 594 nm.

Red needle-shaped single crystals have been obtained by slow diffusion of ether vapour into a dichloromethane solution of **2aTPP$_2$** at room temperature.

2cTPP$_2$: the same synthetic procedure described for **5a** was followed starting from 5 mg of **2c** (5 µmol) and 7.2 mg of **ZnTPP** (10 µmol). Yield: 10.8 mg (88%). ^1H NMR (500 MHz, $CDCl_3$, 25 °C): δ = 8.85 (s, 16H; H_f), 8.17 (d, *J*(H,H) = 6.0 Hz, 16H; H_c), 7.72 (m, 24H; H_d, H_e), 5.81 (s, 4H, H_k), 3.01 (s, 4H, H_j), 2.31 (s, 12H,), 1.44 (s, 12H); ^{13}C NMR (125 MHz, $CDCl_3$, 25 °C, from HSQC): δ = 134.66 (C_f), 131.53 (C_c), 126.48 (C_d, C_e), 125.19 (C_k), 25.58 (C_q), 20.84 (C_r).

Red needle-shaped single crystals have been obtained by slow diffusion of *n*-hexane into a chloroform solution of **2cTPP$_2$** at room temperature.

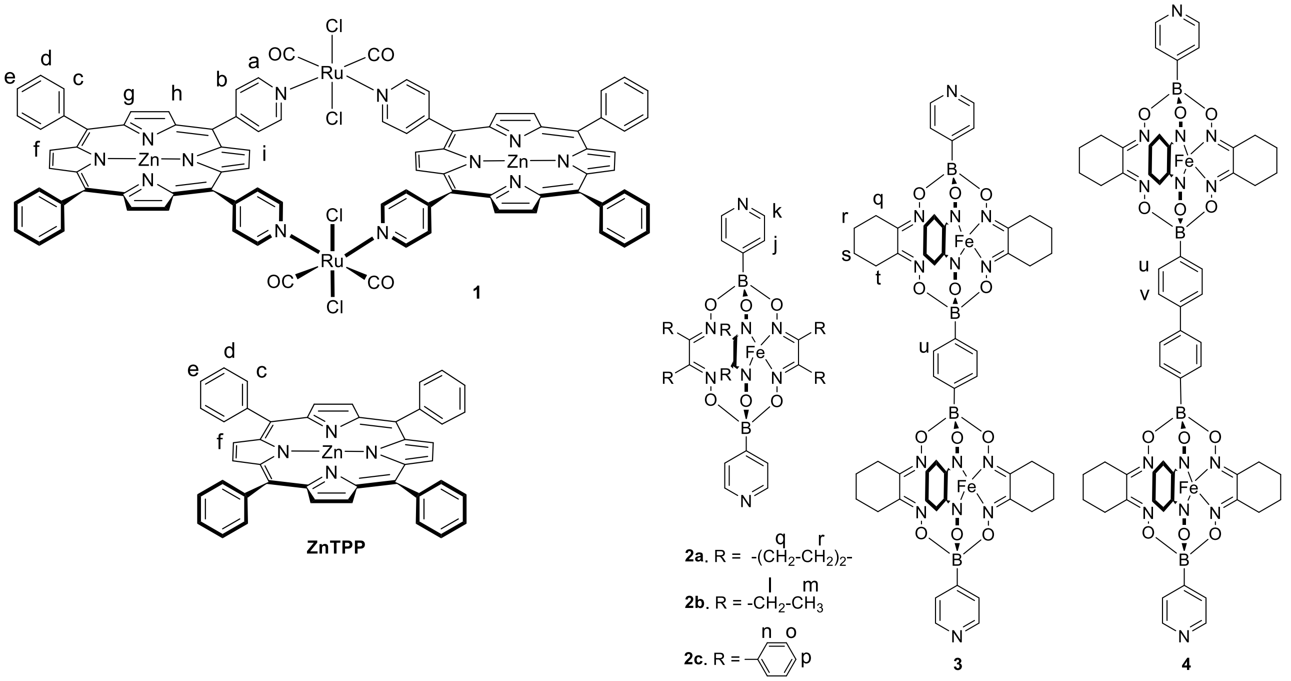

Figure S1. Schematic depiction with labeling of the building blocks: [*t*,*c*,*c*-$RuCl_2(CO)_2$(Zn·4′cisDPyP)]$_2$ (**1**), 4'-dipyridyl-Fe^{II}(clathrochelate) ligands (**2**–**4**), and Zn^{II}-*meso*-tetraphenylporphyrin (**ZnTPP**).

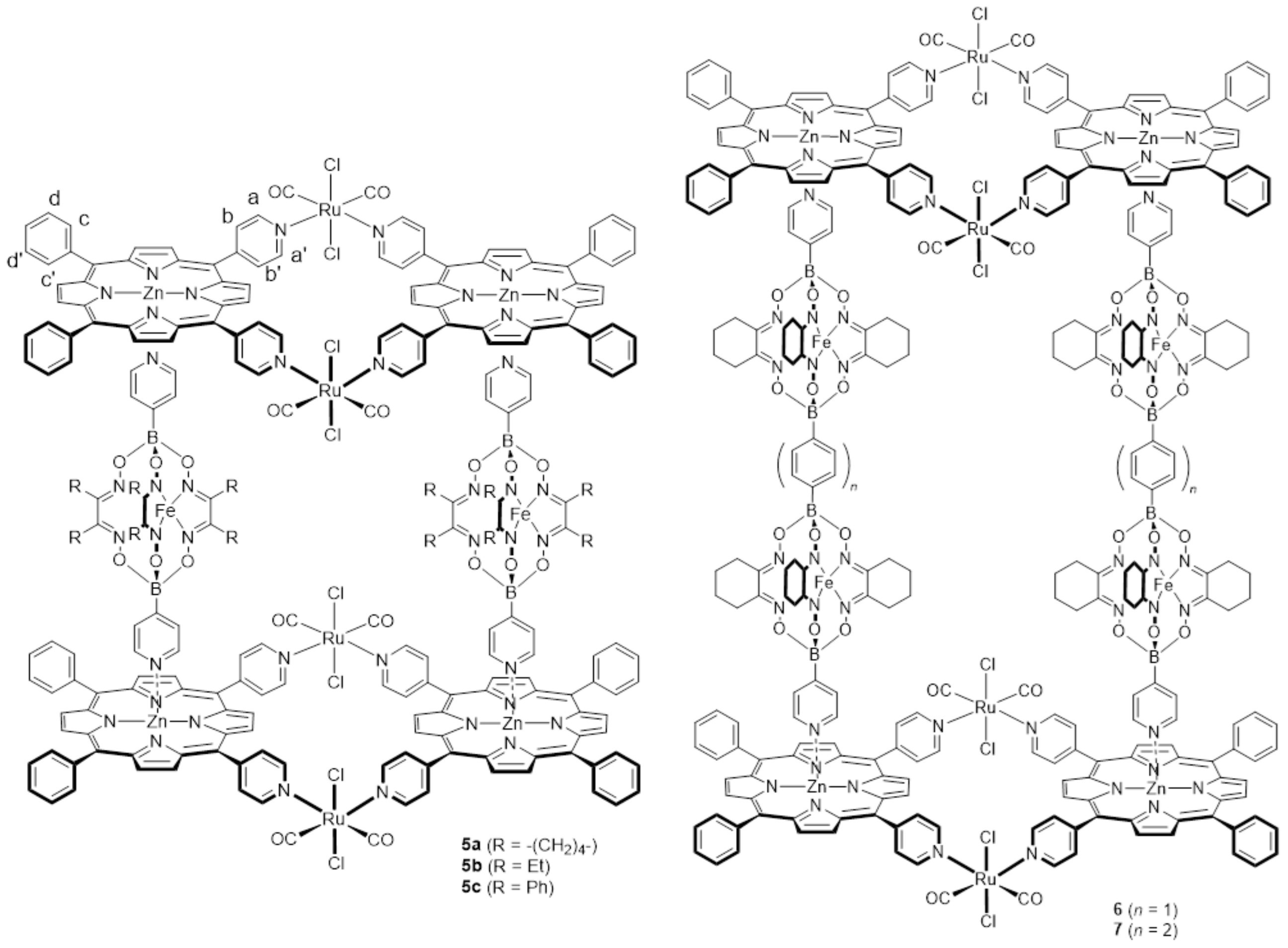

Figure S2. Schematic depiction of the supramolecular assemblies {[*t*,*c*,*c*-$RuCl_2(CO)_2$(Zn·4′cisDPyP)]$_2$}$_2${4′-dipyridyl- Fe_n^{II}(clathrochelate)}$_2$ (*n* = 1: **5a**–**c**, *n* = 2: **6, 7**).

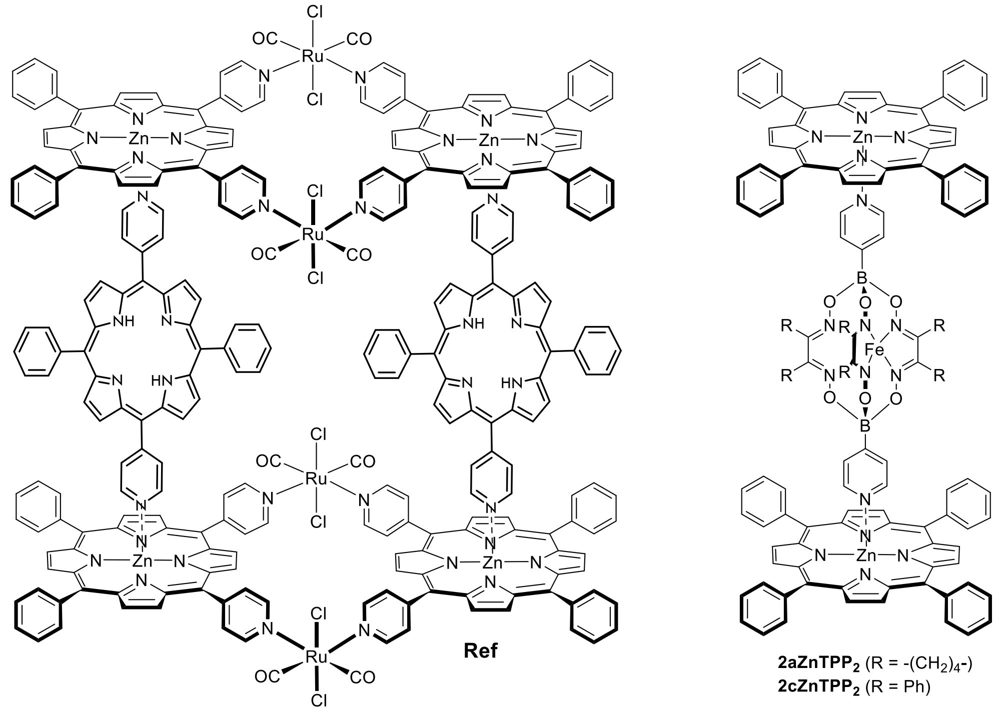

Figure S3. Schematic depiction of the reference assembly {[*t*,*c*,*c*-$RuCl_2(CO)_2$(Zn·4′cisDPyP)]$_2$}$_2${4′transDPyP}$_2$ (**8**)[15] and models {Zn·TPP}$_2${4′-dipyridyl-Fe^{II}(clathrochelate)}$_2$ (**2aTPP₂**, **2cTPP₂**).

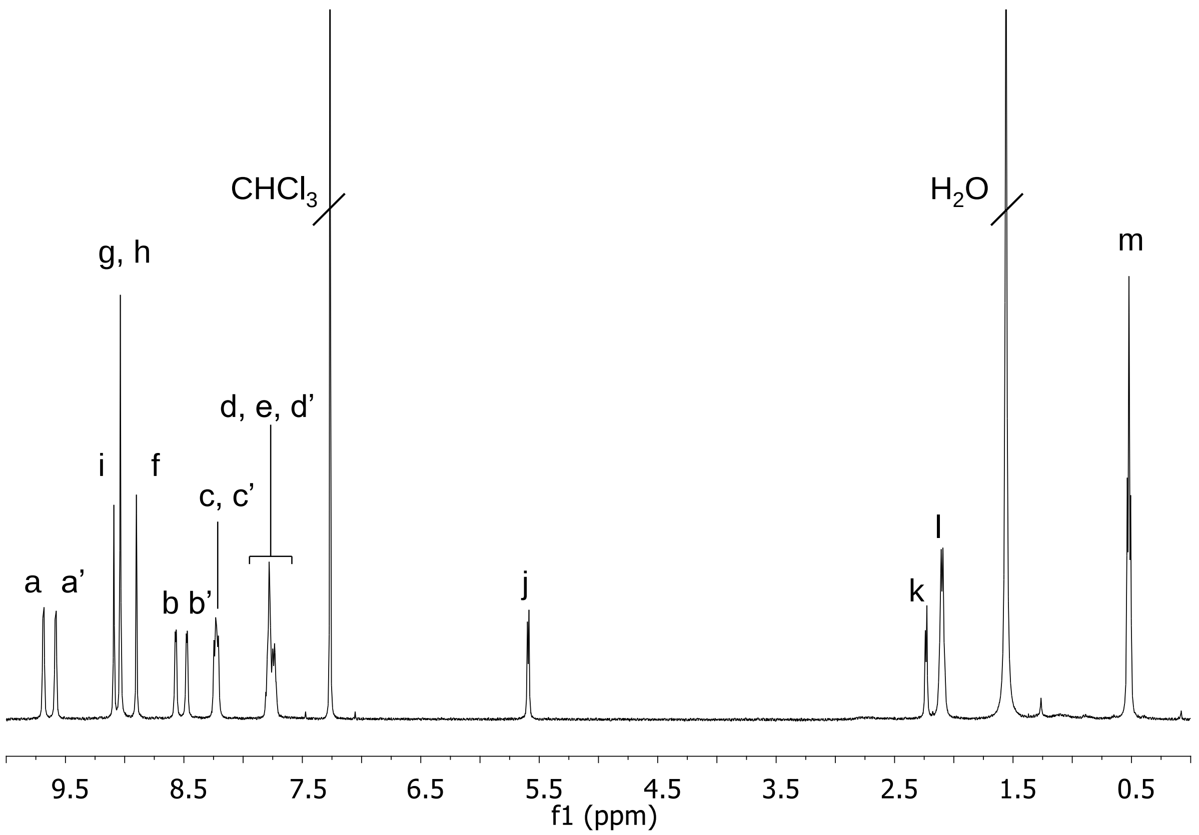

Figure S4. ^{1}H NMR spectrum ($CDCl_3$) of **5b**.

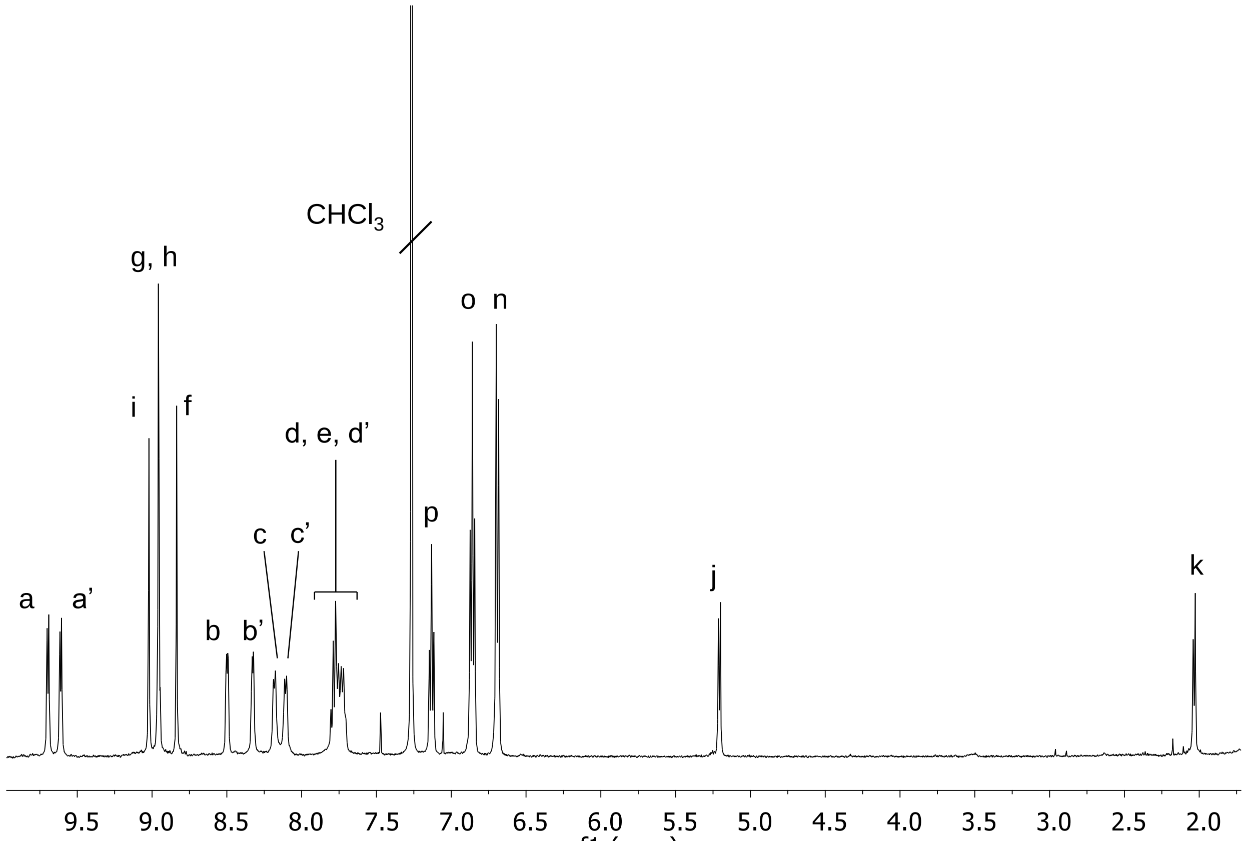

Figure S5. ^{1}H NMR spectrum ($CDCl_3$) of **5c**.

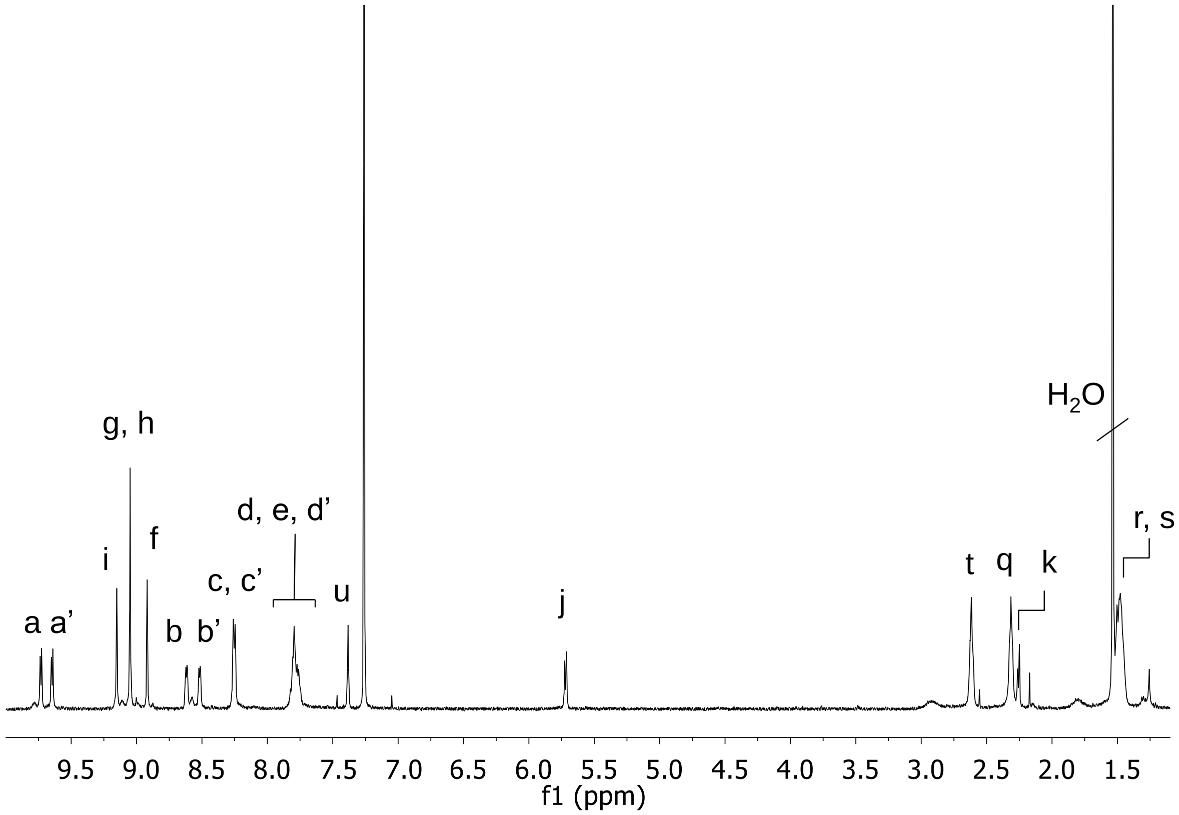

Figure S6. ^{1}H NMR spectrum ($CDCl_3$) of **6**.

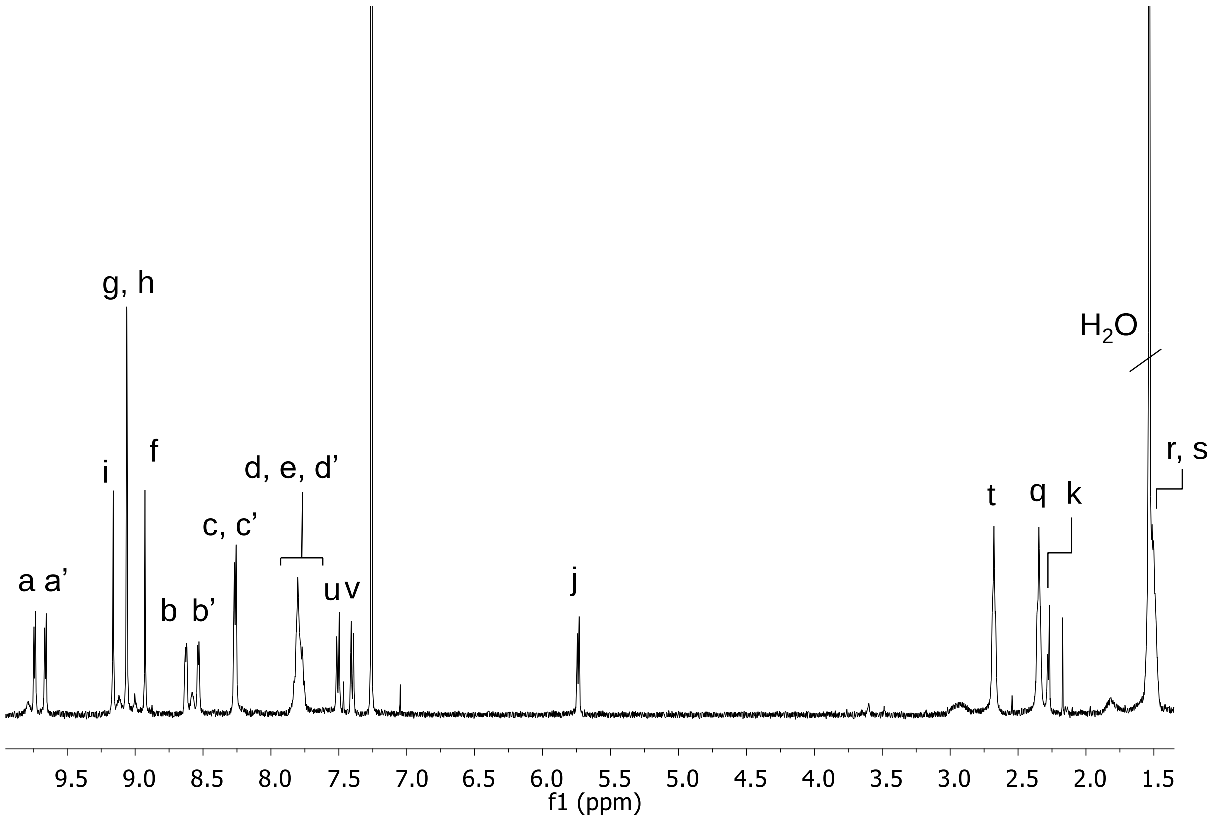

Figure S7. ^{1}H NMR spectrum ($CDCl_3$) of **7**.

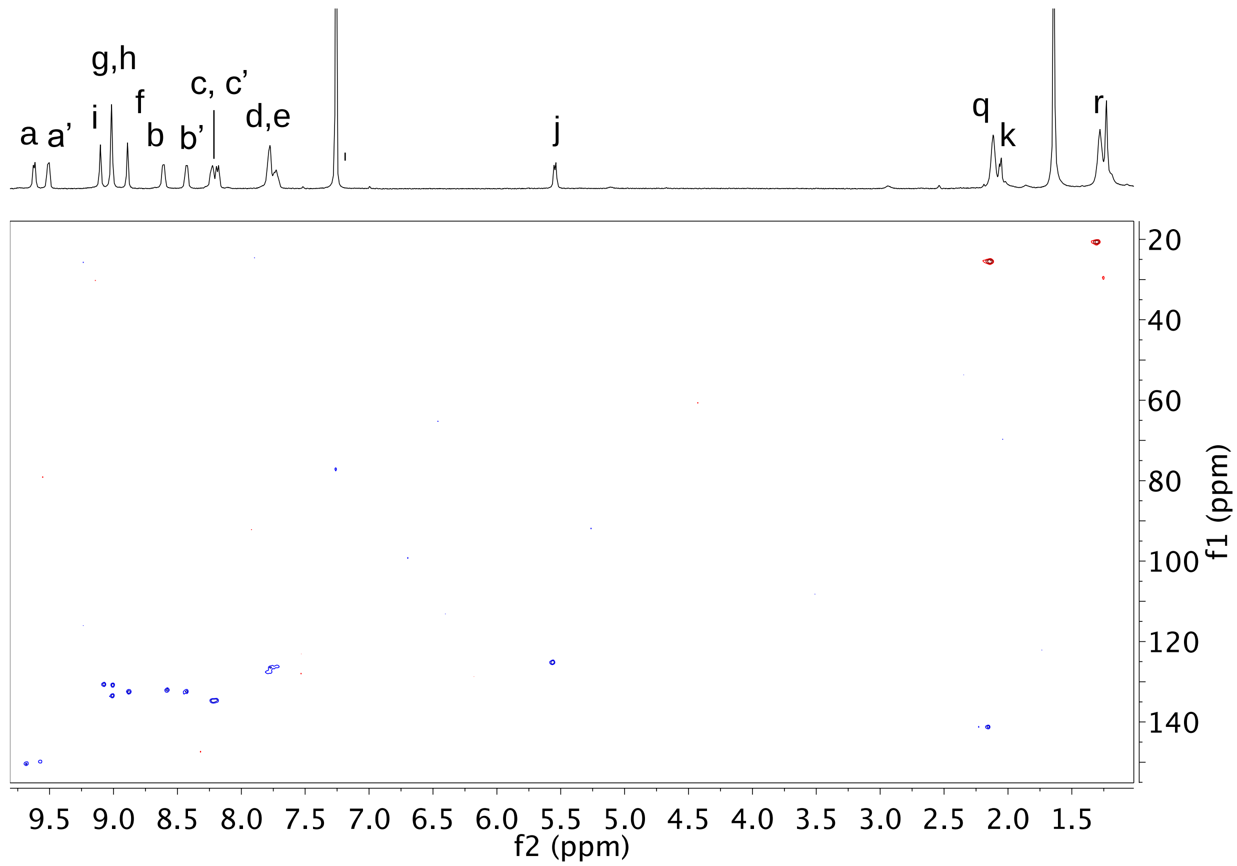

Figure S8. ^{1}H-^{13}C HSQC spectrum ($CDCl_3$) of **5a**.

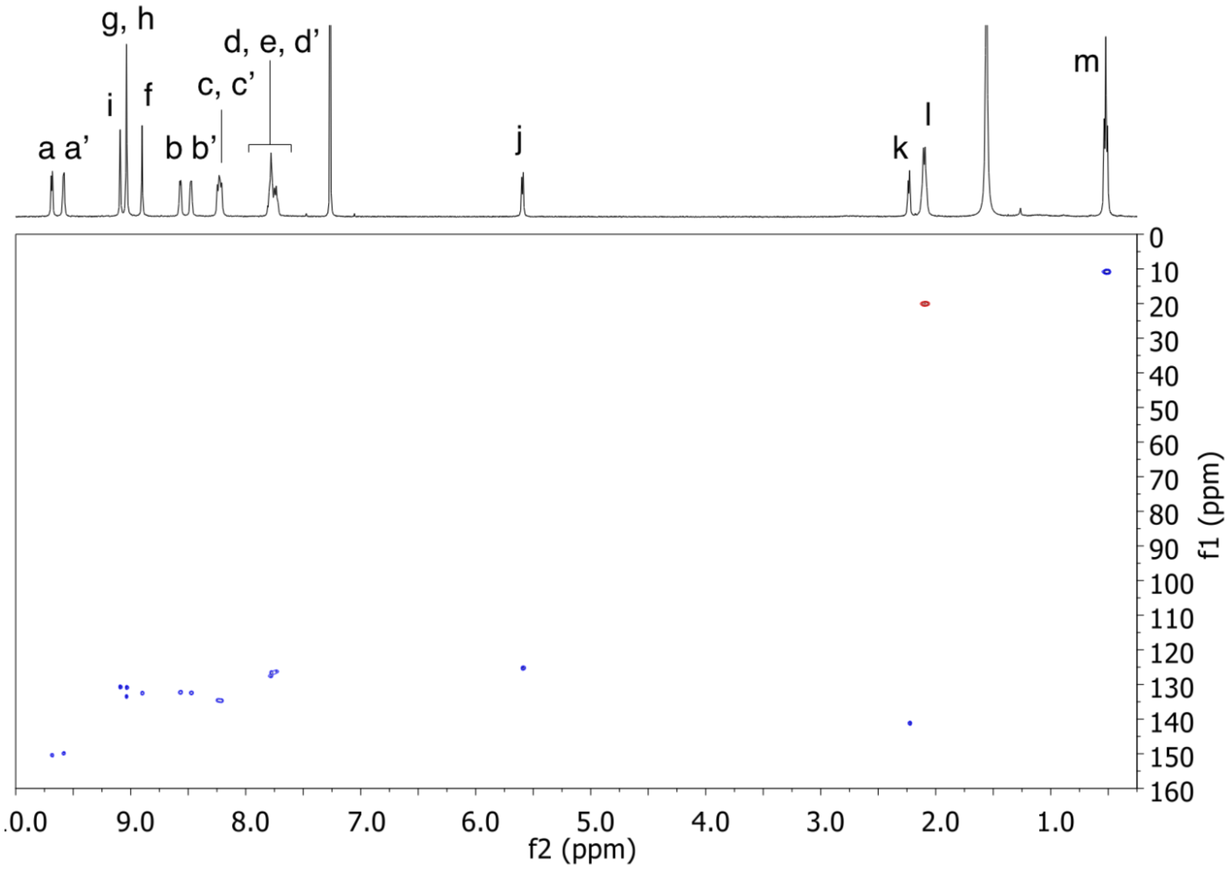

Figure S9. ^{1}H-^{13}C HSQC spectrum ($CDCl_3$) of **5b**.

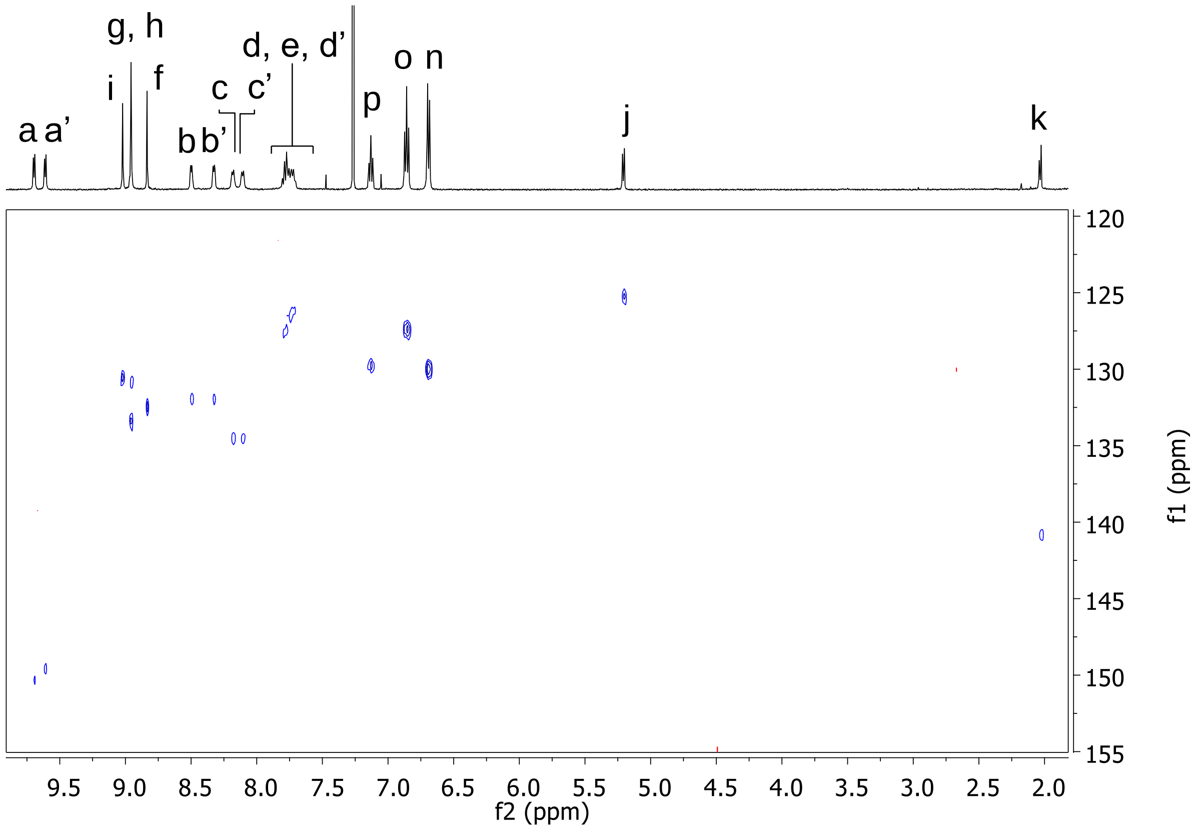

Figure S10. ^{1}H-^{13}C HSQC spectrum ($CDCl_3$) of **5c**.

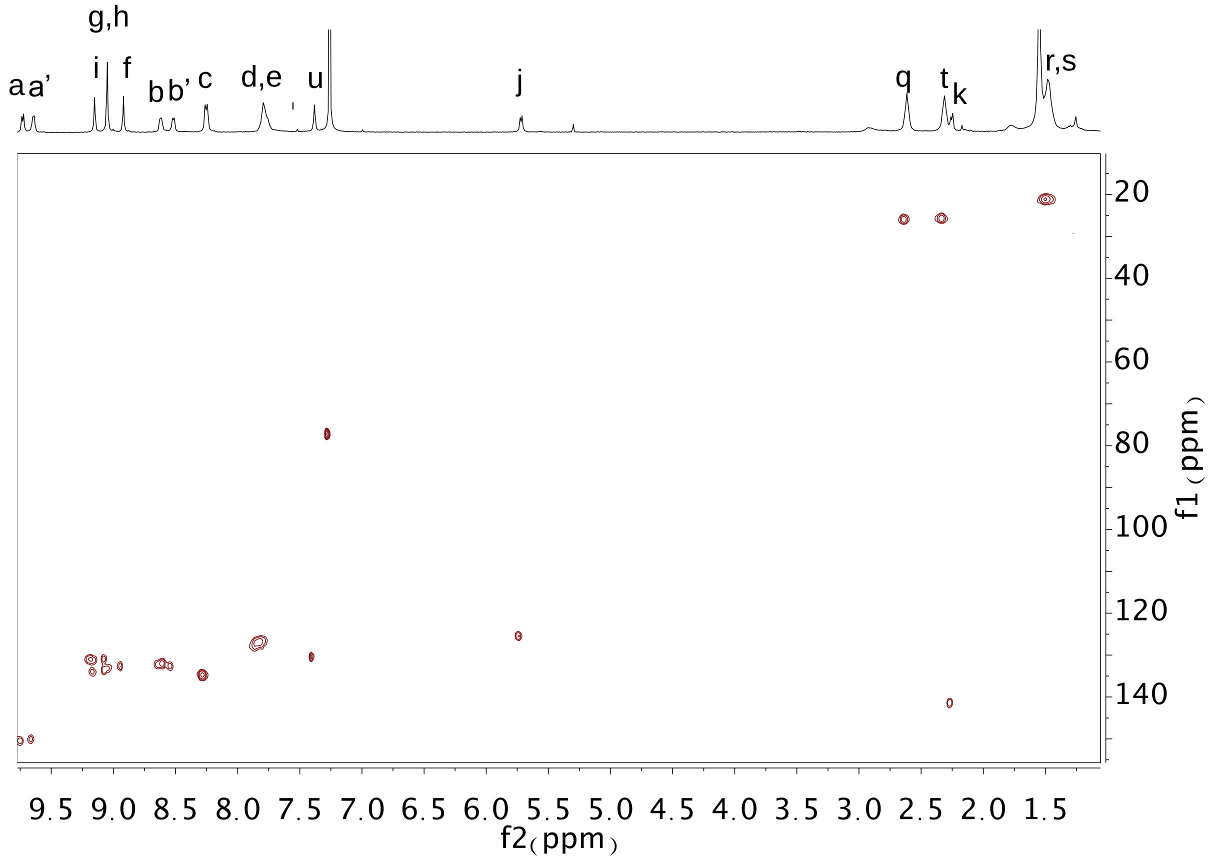

Figure S11. ^{1}H-^{13}C HSQC spectrum ($CDCl_3$) of **6**.

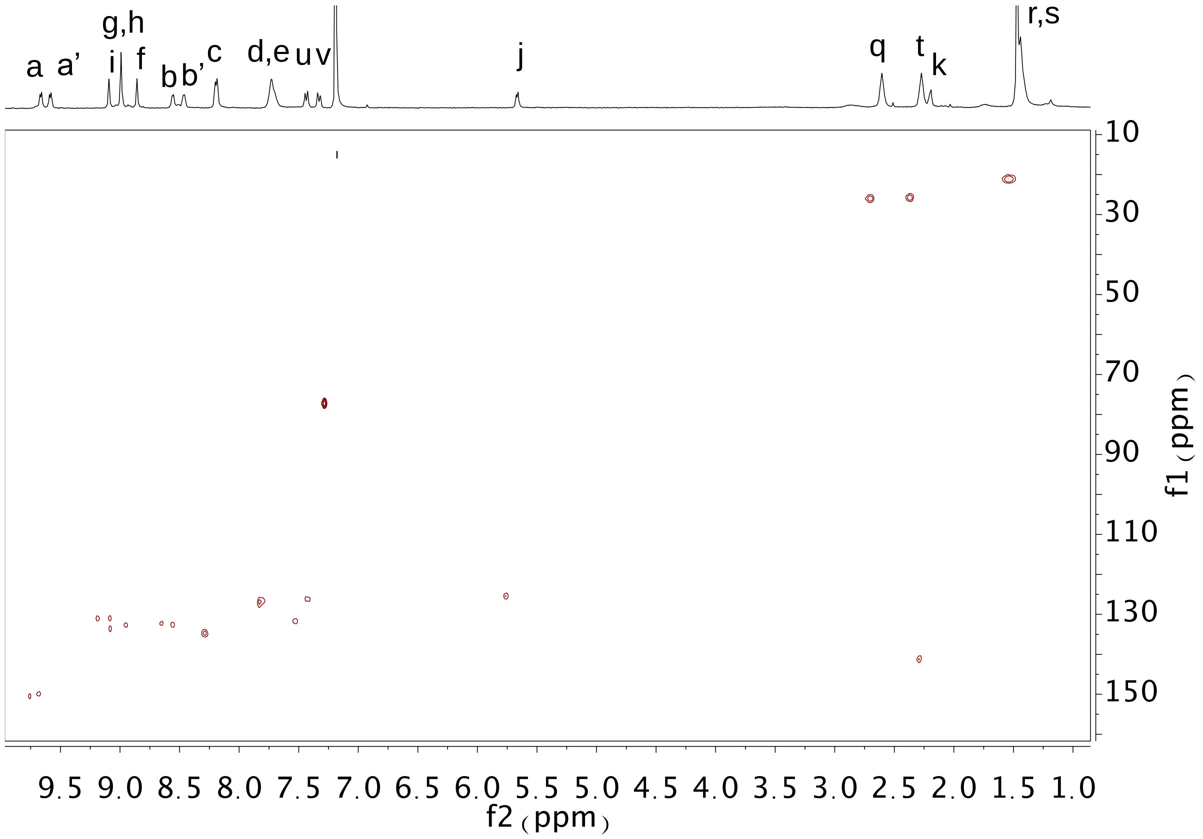

Figure S12. ^{1}H-^{13}C HSQC spectrum ($CDCl_3$) of **7**.

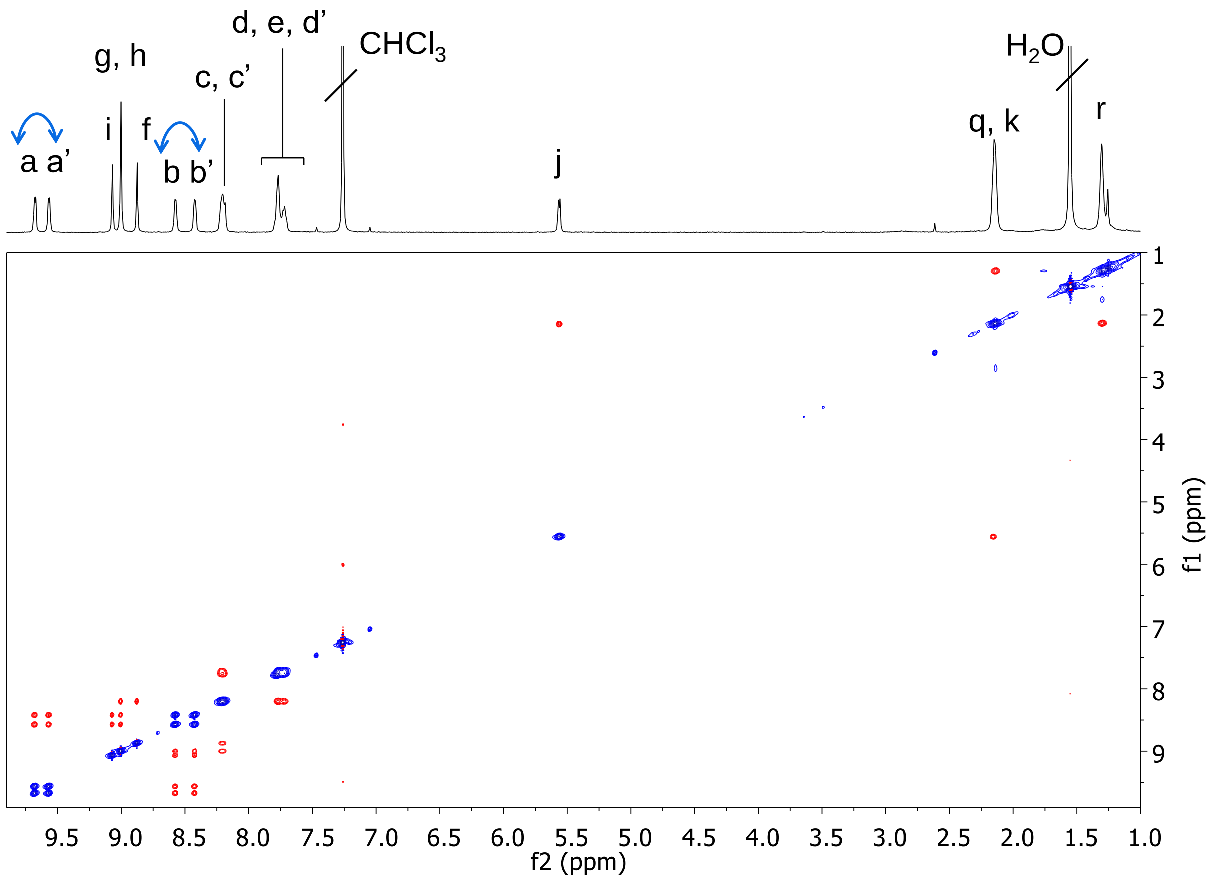

Figure S13. ^{1}H-^{1}H ROESY spectrum ($CDCl_3$) of **5a**.

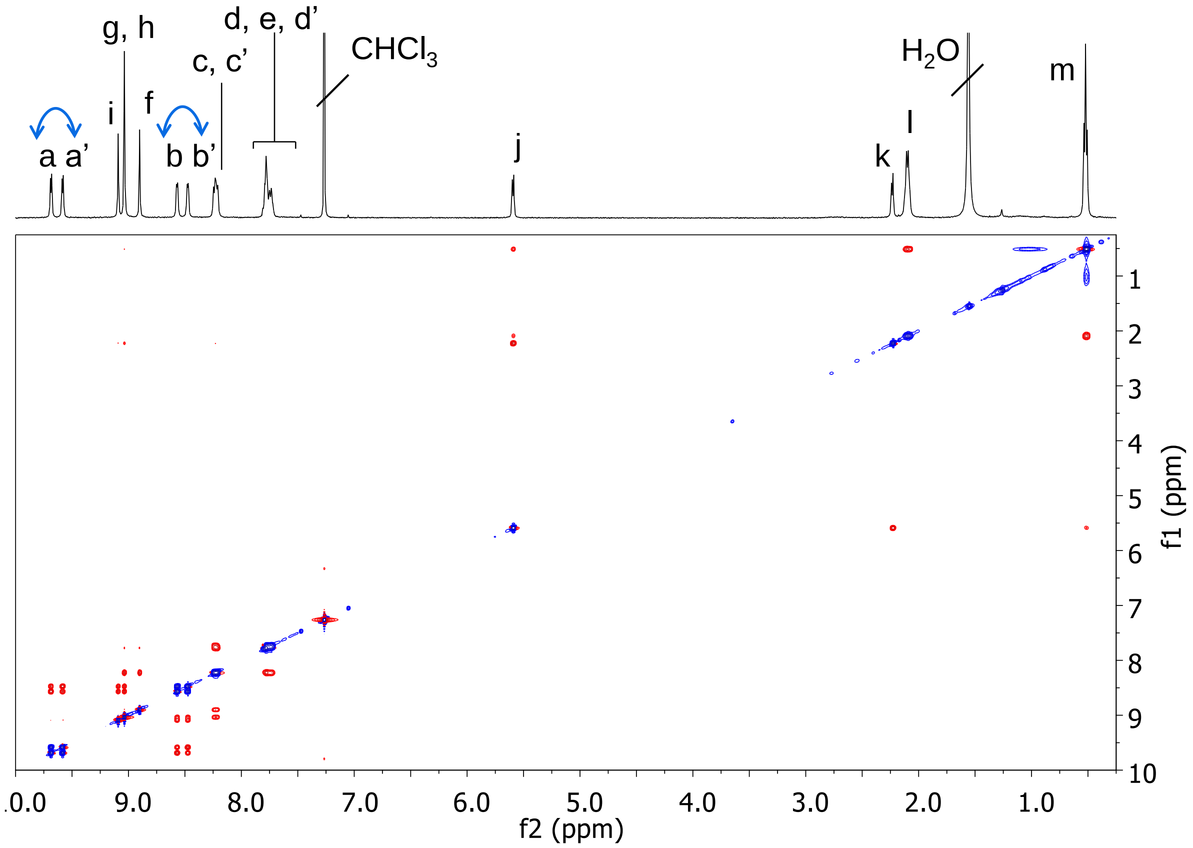

Figure S14. ^{1}H-^{1}H ROESY spectrum ($CDCl_3$) of **5b**.

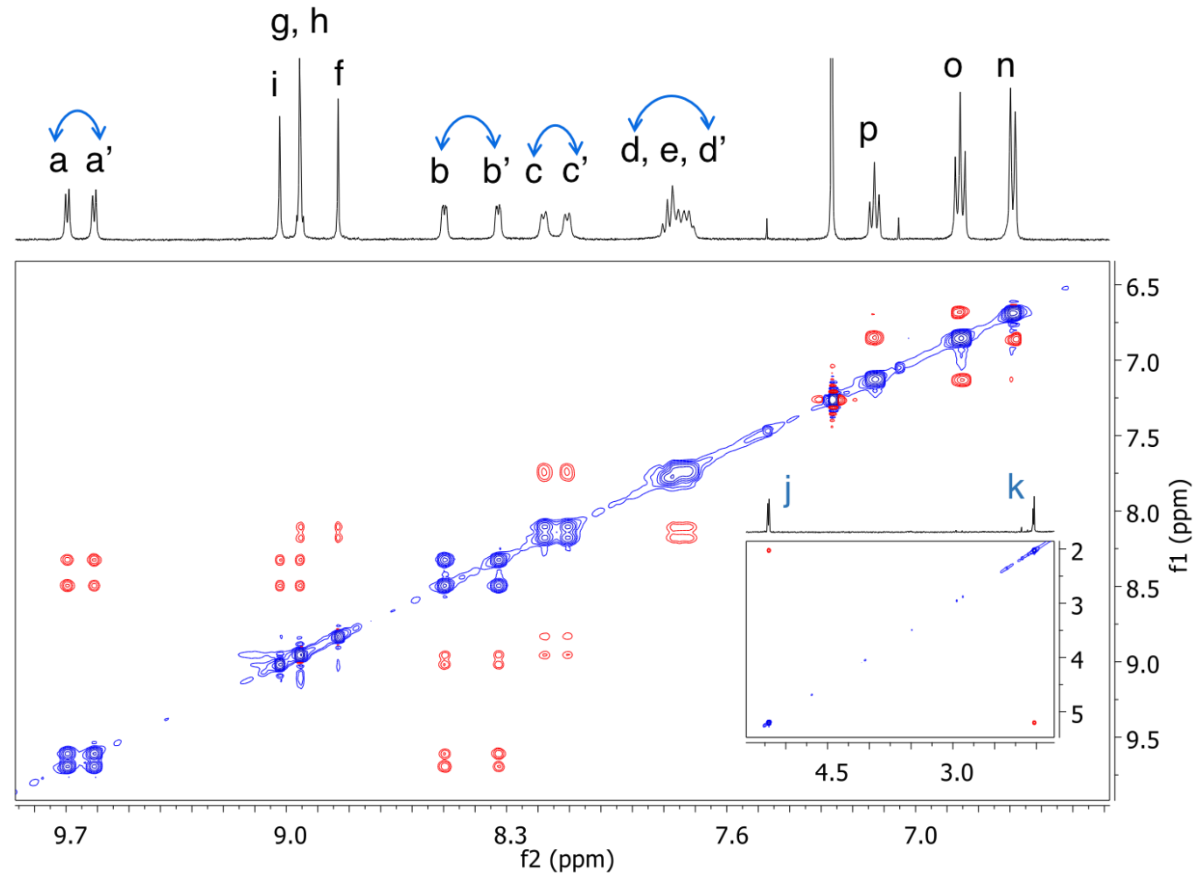

Figure S15. ^{1}H-^{1}H ROESY spectrum ($CDCl_3$) of **5c**.

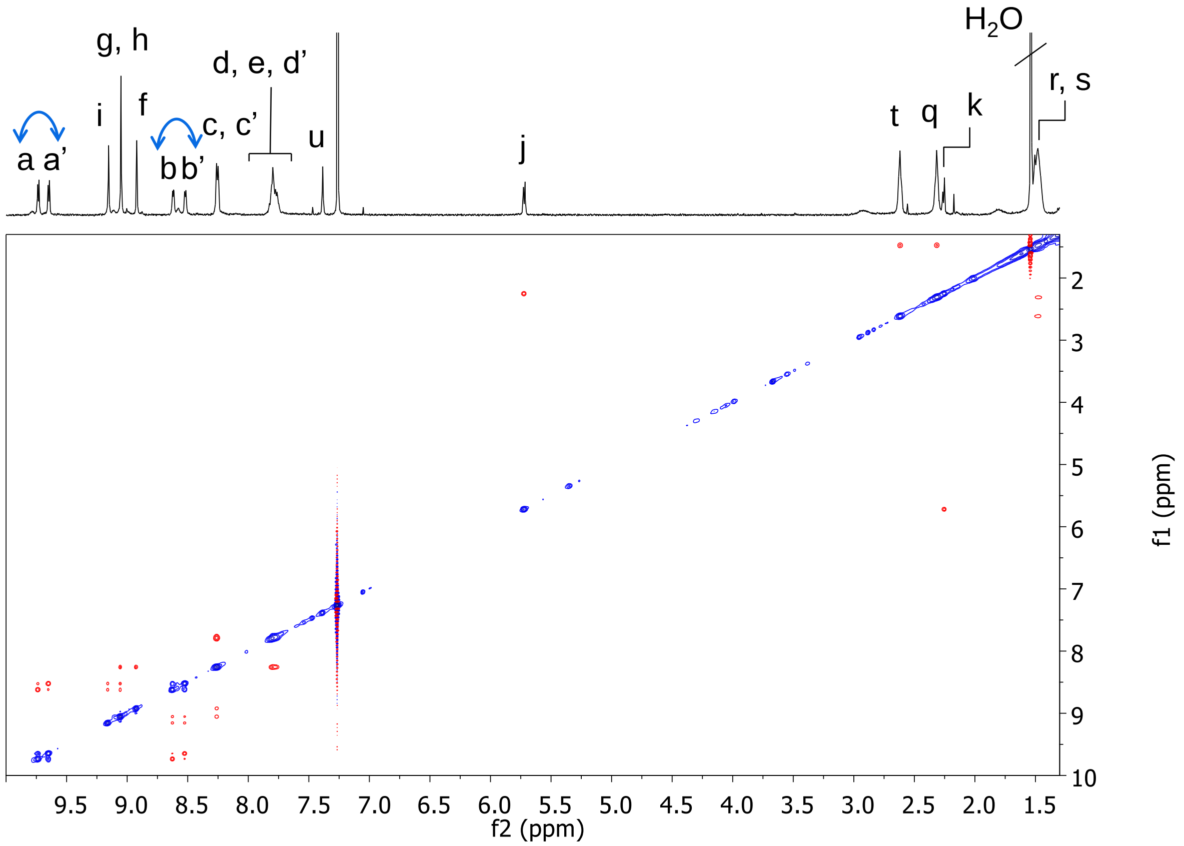

Figure S16. ^{1}H-^{1}H ROESY spectrum ($CDCl_3$) of **6**.

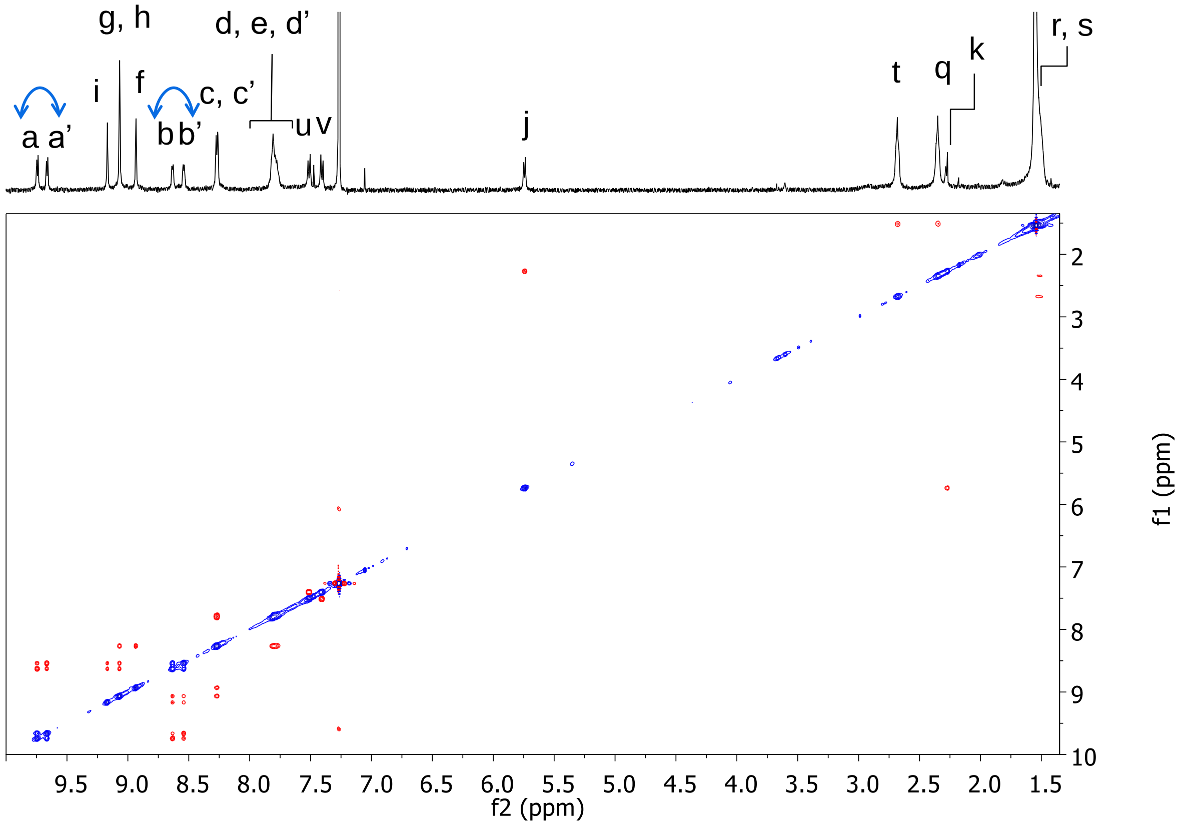

Figure S17. ^{1}H-^{1}H ROESY spectrum ($CDCl_3$) of **7**.

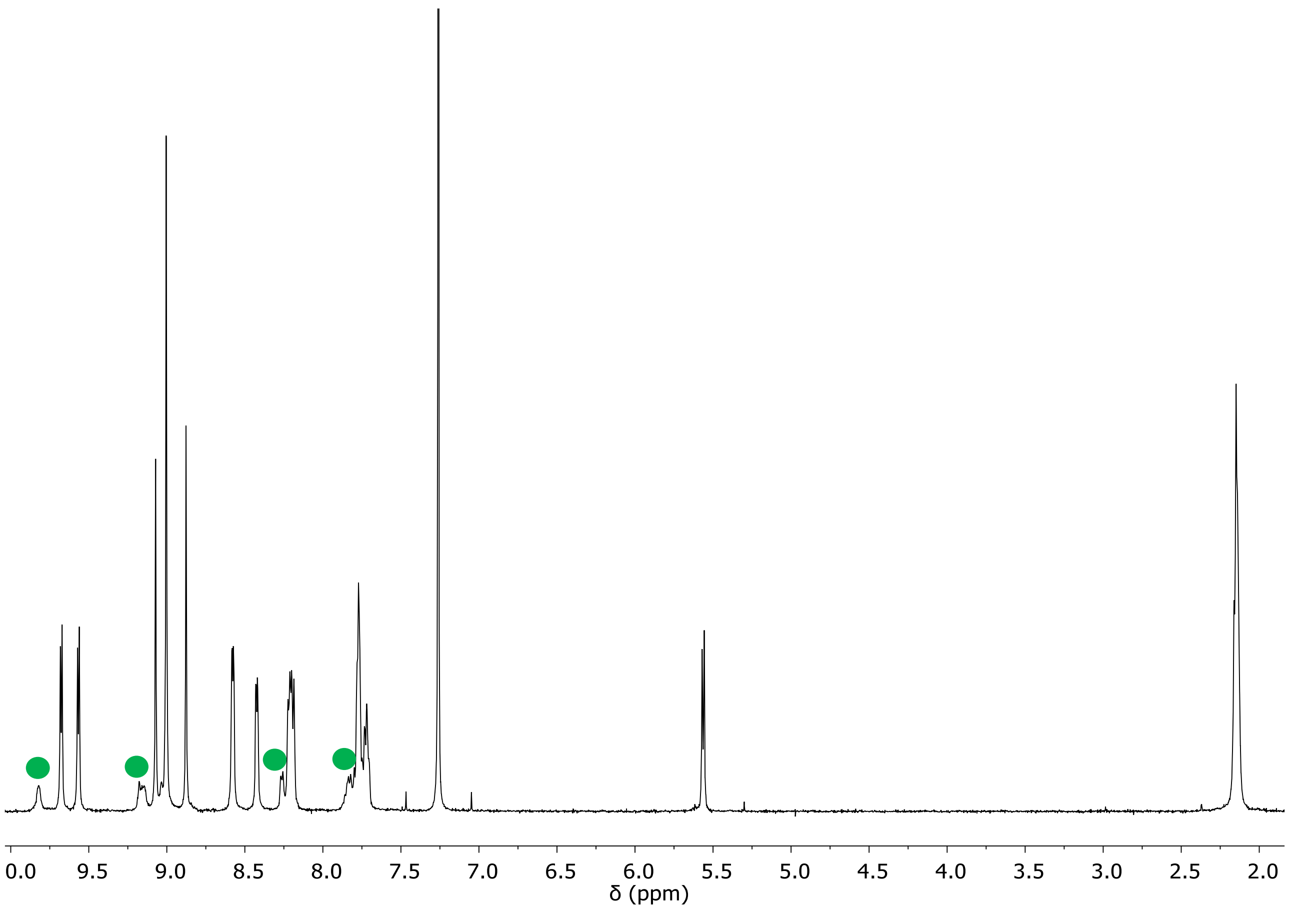

Figure S18. ^{1}H NMR ($CDCl_3$) of **5a** with an excess of **1** (green dots).

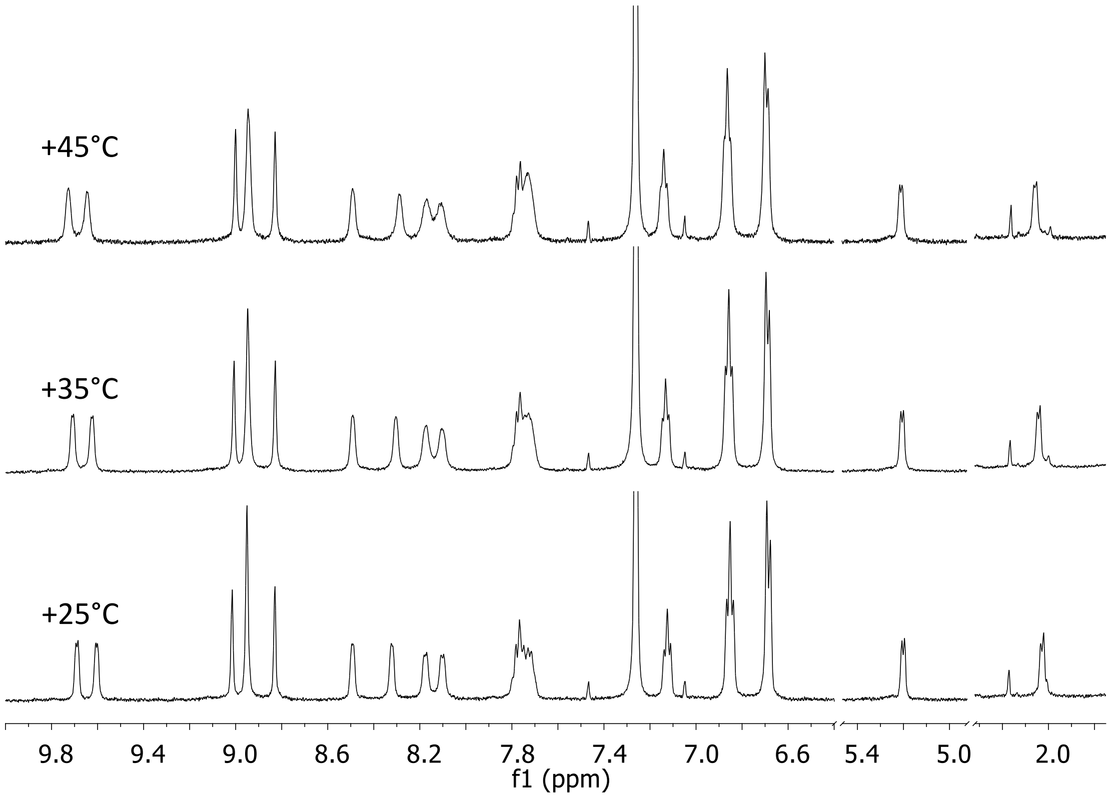

Figure S19. VT ^{1}H NMR ($CDCl_3$) of **5c** between +25 ÷ +45 °C.

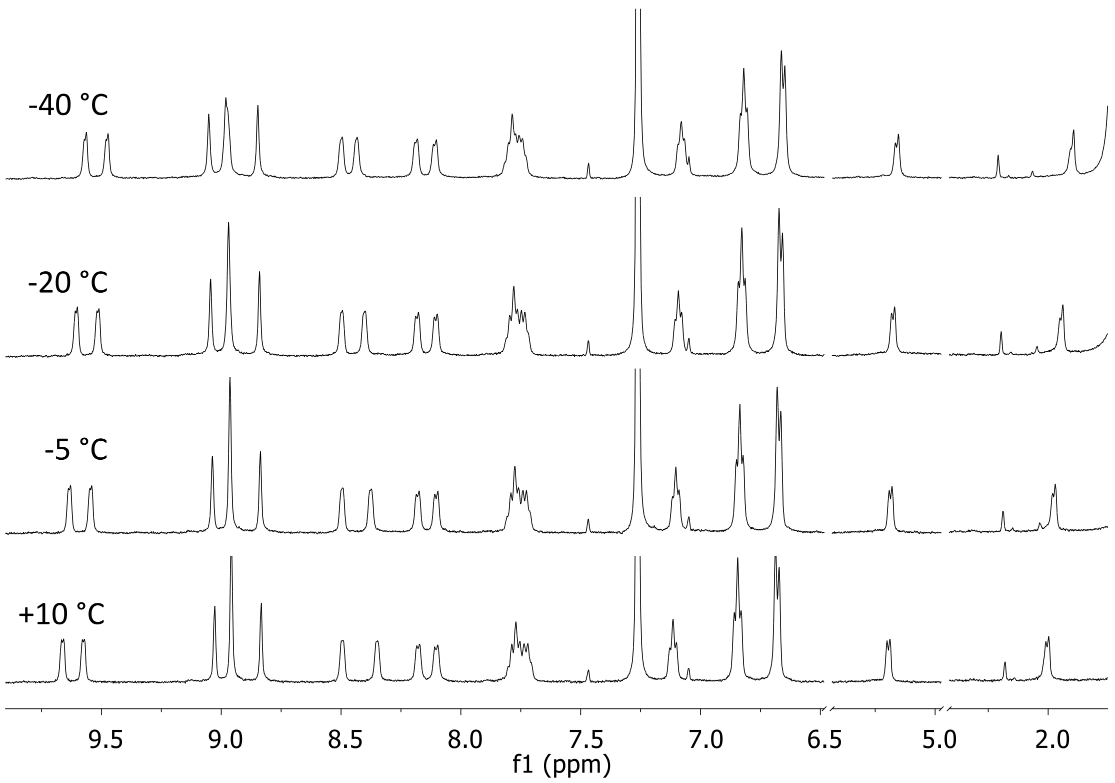

Figure S20. VT ^{1}H NMR ($CDCl_3$) of **5c** between +10 ÷ –40 °C.

Mass analysis

Over the years, we learned that the characterization of neutral assemblies of porphyrin obtained by axial coordination of polypyridyl ligands to Zn-porphyrin Ru-metallacyclic platforms, like **1**, poses several challenges.[11,12,53,54] In specific, the assemblies get dismantled by (successive) protonation of the pyridyl connectors, and only the molecular ion peaks corresponding to these latter can be observed, while the connecting Zn^{II}-porphyrin platform **1** is insufficiently volatile to be even detected. Likewise, numerous attempts to obtain mass data performed with the indoor electrospray instrument, or elsewhere MALDI facilities, on **5a-c**, **6** and **7** were unsuccessful. Invariably, only the molecular ion peaks corresponding to the mono- and di-protonated metalloligands were detected, as examples see Figures S21, S22 and S31.

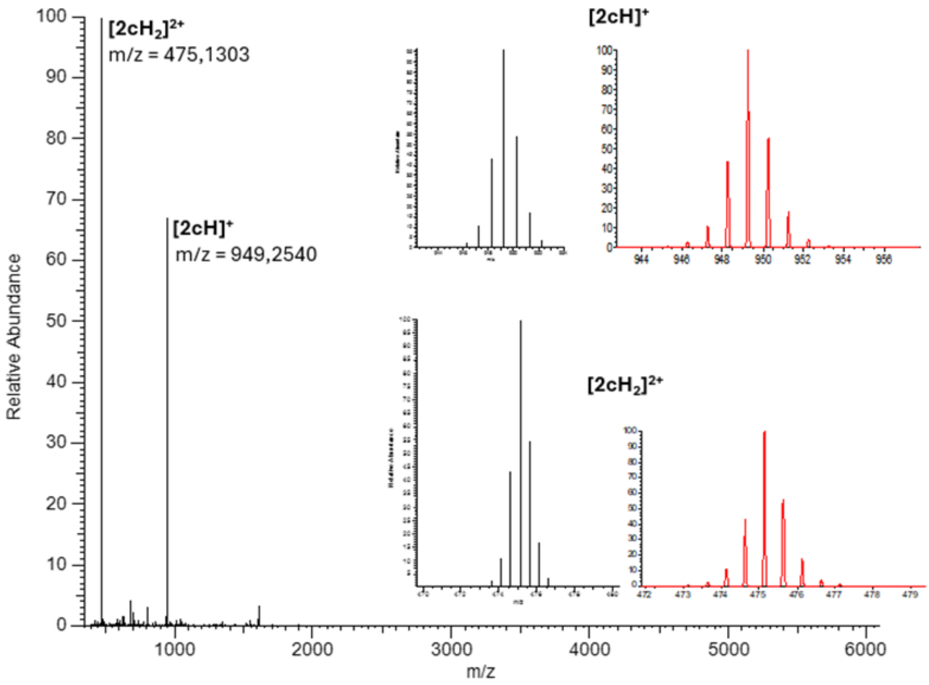

Figure S21. ESI-MS (m/z), positive mode, of **5c**. Only the molecular ion peaks corresponding to the mono- and di-protonated metalloligand **2c** can be detected – the insets show the corresponding experimental and calculated isotopic distributions.

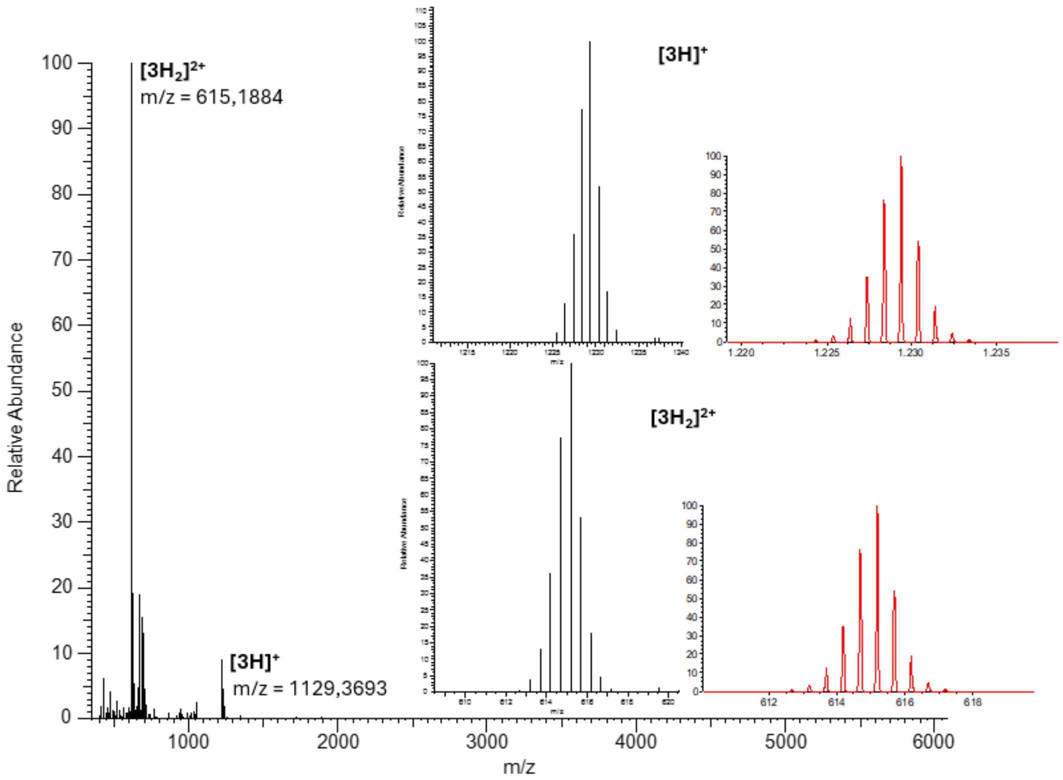

Figure S22. ESI-MS (m/z), positive mode, of **6**. Only the molecular ion peaks corresponding to the mono- and di-protonated metalloligand **3** can be detected – the insets show the corresponding experimental and calculated isotopic distributions.

DOSY experiments

The diffusion coefficients (D_t) reported in Table S1 represent the average of at least five different D_t values, calculated from the analysis of 1D DOSY spectra. Each D_t was determined by fitting the proton signal decay to a mono-exponential function (Figure S20) using the Stejskal-Tanner equation.[70] The hydrodynamic radius (r_H) for each species was then calculated using the Stokes-Einstein equation, assuming the spherical approximation. This approximation was considered reasonable for our systems, as the ratio between the longest and largest axes in the structures does not exceed 1.5.[77-80]

Table S1. Experimental diffusion coefficient values D_t determined from the DOSY experiments. Hydrodynamic radius r_H and hydrodynamic volume V_H calculated from Stokes-Einstein equation.

	D_t[a]	r_H (Å)[b]	V_H (Å^3)[c]
**1**	5.58 ± 0.03	7.3	1630
**2a**	8.41 ± 0.03	4.8	463
**2b**	8.44 ± 0.06	4.8	463
**2c**	6.41 ± 0.08	6.3	1047
**3**	6.17 ± 0.04	6.6	1204
**4**	5.58 ± 0.08	7.3	1630
**5a**	3.70 ± 0.03	11.0	5575
**5b**	3.67 ± 0.07	11.1	5729
**5c**	3.71 ± 0.08	11.0	5575
**6**	3.36 ± 0.03	12.1	7420
**7**	3.13 ± 0.04	13.0	9200

(a) D_t /10^{-10} m^2 s^{-1}. (b) Calculated from Stokes-Einstein ($\eta_{chloroform}$ = 5.37 x10^{-4} N m^{-2} s, at 25 °C). (c) $V_H = {}^4/_3 \pi r_H^3$.

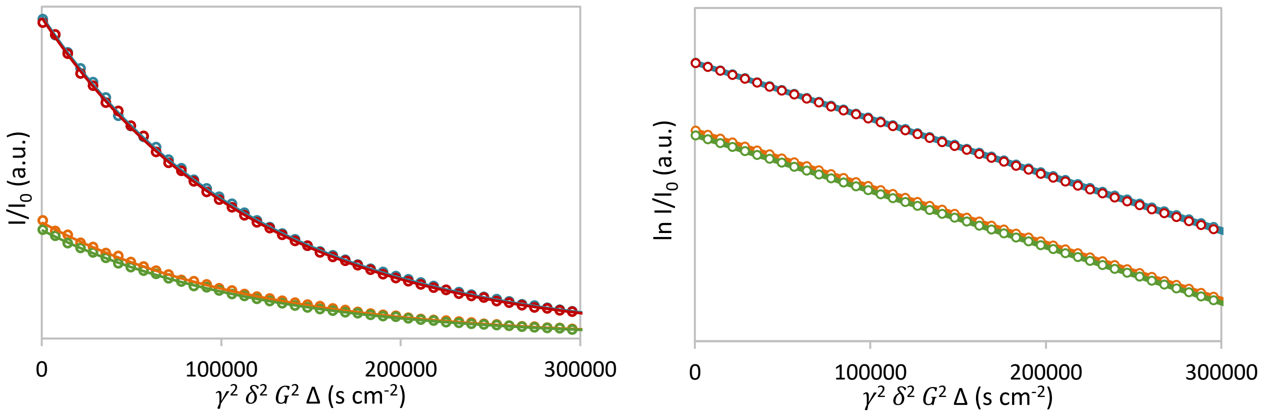

Figure S23. ^{1}H DOSY analysis for **5a**: mono-exponential fit of some proton signal decays (left), and linearization of these decays (right).

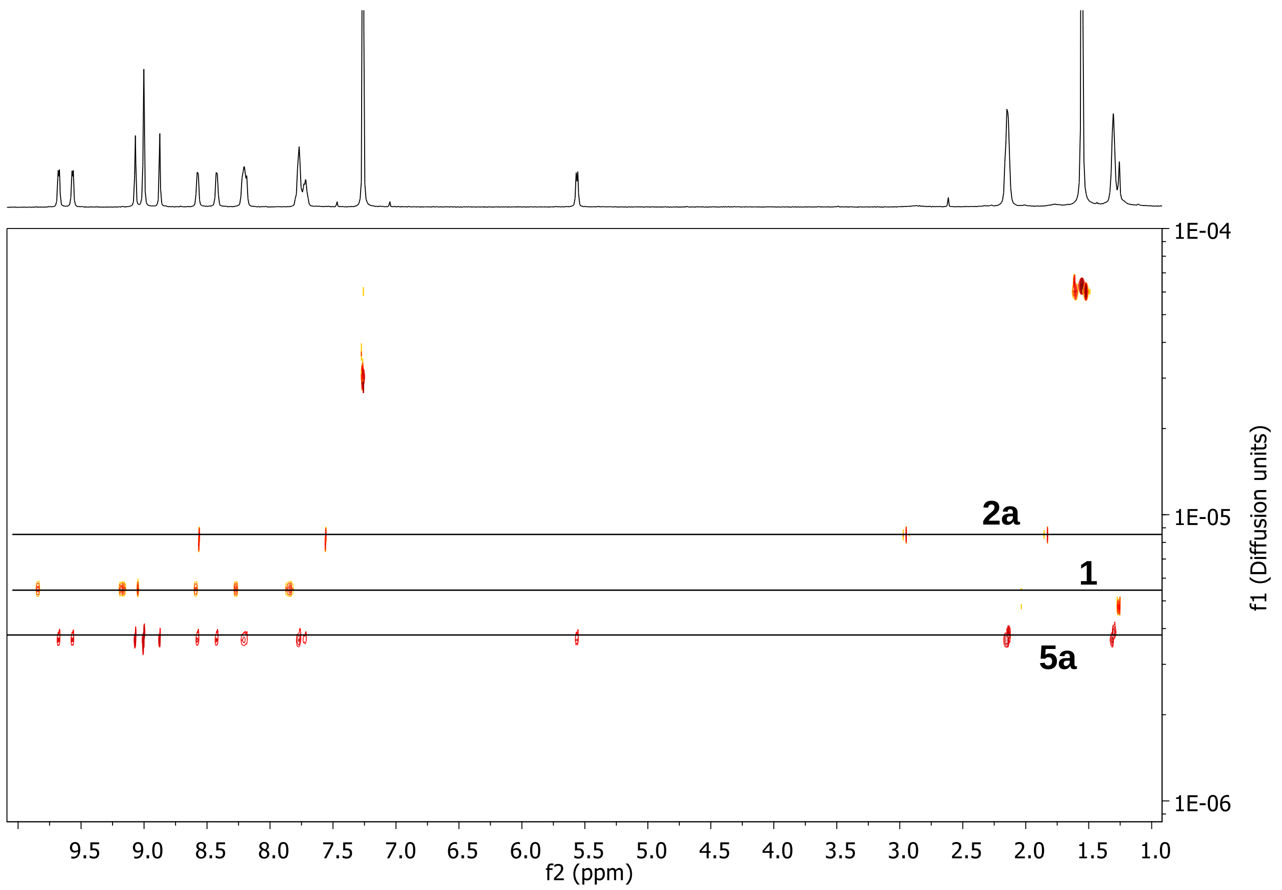

Figure S24. Overlay of the 2D ^{1}H DOSY spectra ($CDCl_3$) of Fe^{II}-metalloligand **2a**, Zn^{II}-porphyrin platform **1**, and assembly **5a**; only the ^{1}H trace of **5a** is reported in the horizontal axis.

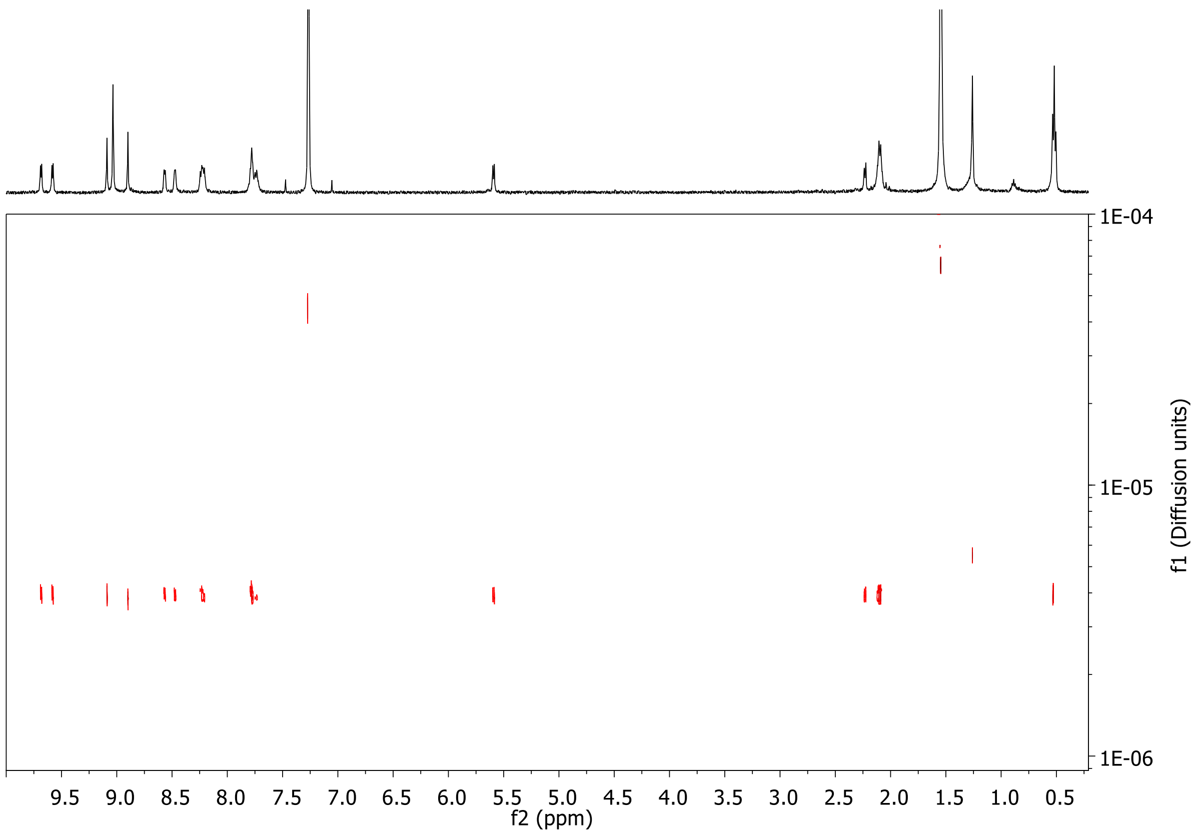

Figure S25. 2D ^{1}H DOSY spectra ($CDCl_3$) of **5b**.

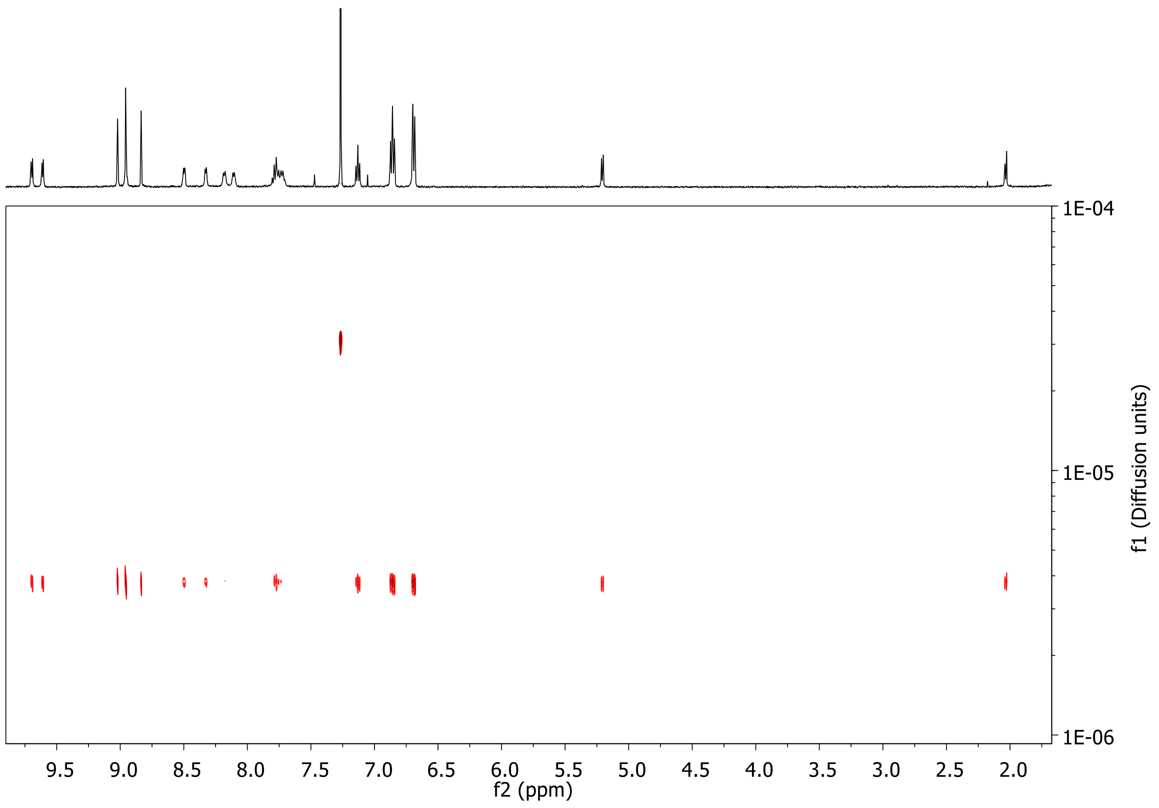

Figure S26. 2D ^{1}H DOSY spectra ($CDCl_3$) of **5c**.

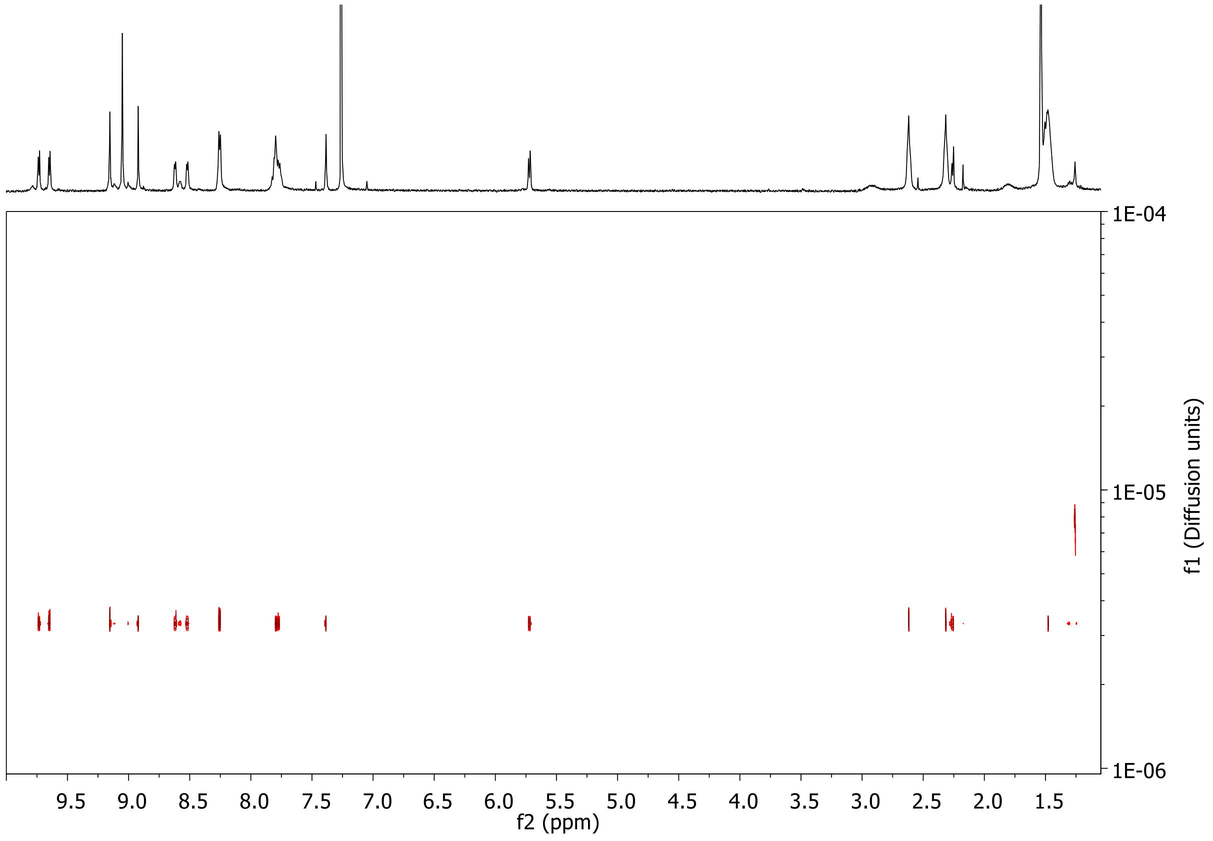

Figure S27. 2D ^{1}H DOSY spectra ($CDCl_3$) of **6**.

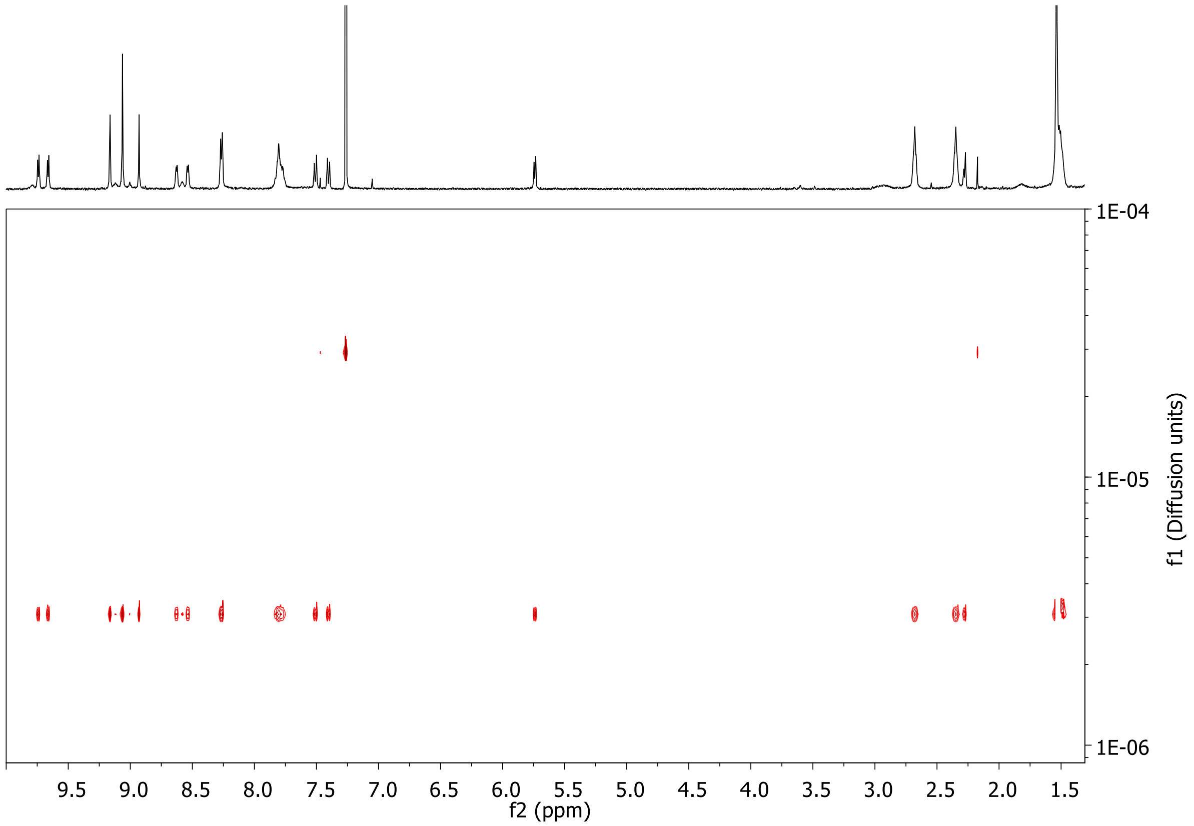

Figure S28. 2D ^{1}H DOSY spectra ($CDCl_3$) of **7**.

Self-sorting experiments.

The study on mixtures of **1**, **2a** and **2c** (**mix1**) was performed by following a single procedure: 0.52 mg of **2a** ($0.8x10^{-3}$ mmol) and 0.76 mg of **2c** ($0.8x10^{-3}$ mmol) were in 0.7 mL of $CDCl_3$ and the ligand stoichiometric ratio was checked by ^1H NMR; 3 mg of **1** ($1.6x10^{-3}$ mmol) were then added and the resulting homogeneous solution was monitored over time by ^1H NMR spectroscopy.

The study on mixtures of **1**, **2a** and **3** (**mix2**) followed three parallel procedures, by keeping the **1**/**2a**/**3** ratio fixed at 2/1/1:

1) 0.52 mg of **2a** ($0.8x10^{-3}$ mmol) and 0.98 mg of **3** ($0.8x10^{-3}$ mmol) were dissolved in 0.7 mL of $CDCl_3$ and the ligand stoichiometric ratio was checked by ^1H NMR; 3 mg of **1** ($1.6x10^{-3}$ mmol) were then added. The resulting homogeneous solution was monitored over time by ^1H NMR spectroscopy.
2) 3.50 mg of **5a** ($7.1x10^{-4}$ mmol) and 4.31 mg of **6** ($7.1x10^{-4}$ mmol) were dissolved in 1 mL of $CDCl_3$ and the resulting homogeneous solution was monitored over time by ^1H NMR spectroscopy.
3) 3.51 mg of **1** ($1.8x10^{-3}$ mmol), 0.61 mg of **2a** ($0.9x10^{-3}$ mmol) and 1.15 mg of **3** ($0.9x10^{-3}$ mmol) were dissolved in 0.7 mL of $CDCl_3$ and the resulting homogeneous solution was monitored over time by ^1H NMR spectroscopy.

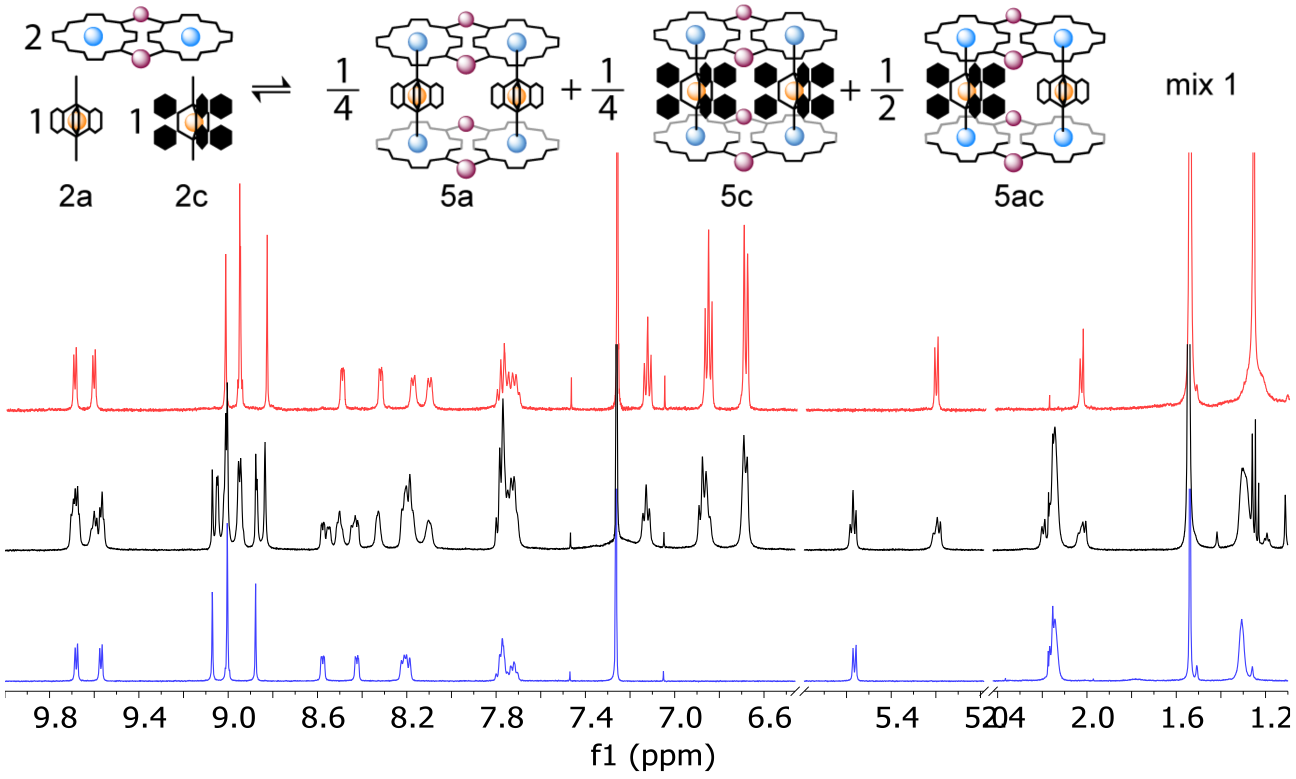

Figure S29. Top: schematic depiction of the equilibrium established by mixing **1**, **2a**, and **2c** in a 2:1:1 ratio (ca. 10^{-3} M in chloroform, at room temperature). Bottom: overlay of the ^{1}H NMR spectra (selected region, $CDCl_3$) of **5c** (red); a 2:1:1 mixture of **1**, **2a**, and **2c** (**mix1**, black); **5a** (blue).

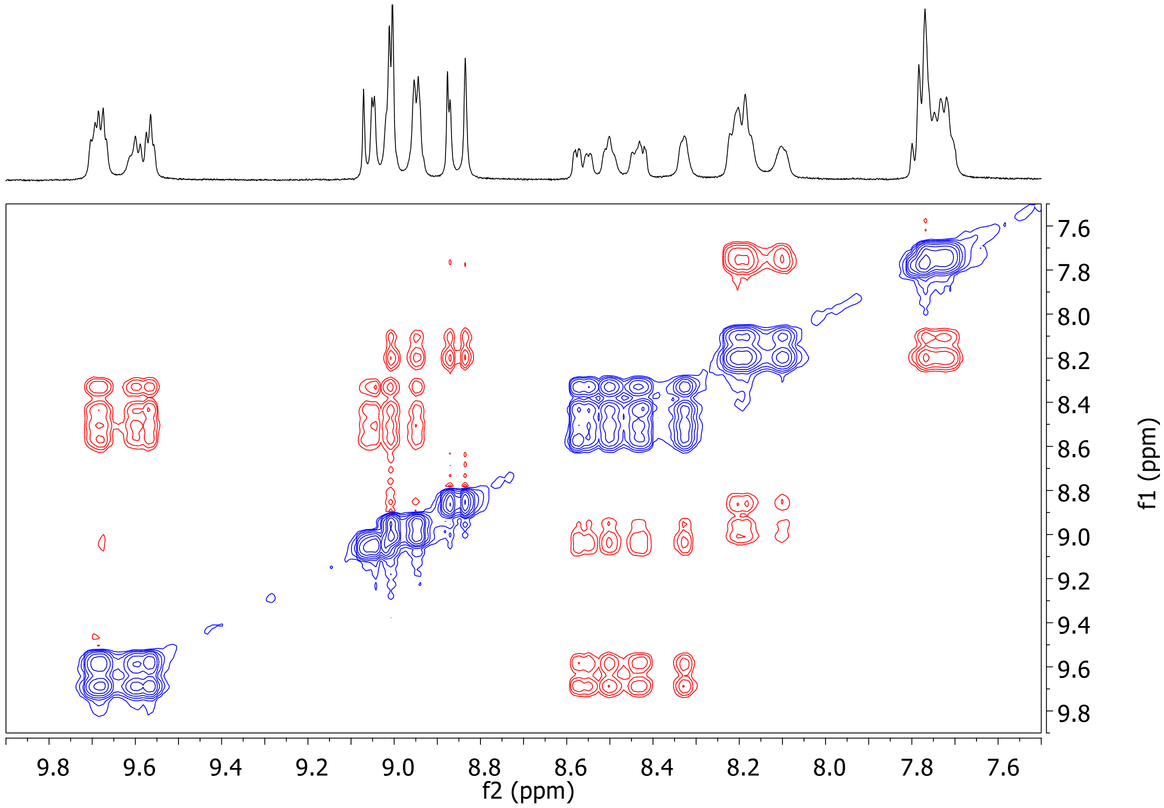

Figure S30. ^{1}H-^{1}H ROESY NMR spectrum ($CDCl_3$, selected region) of **mix1**.

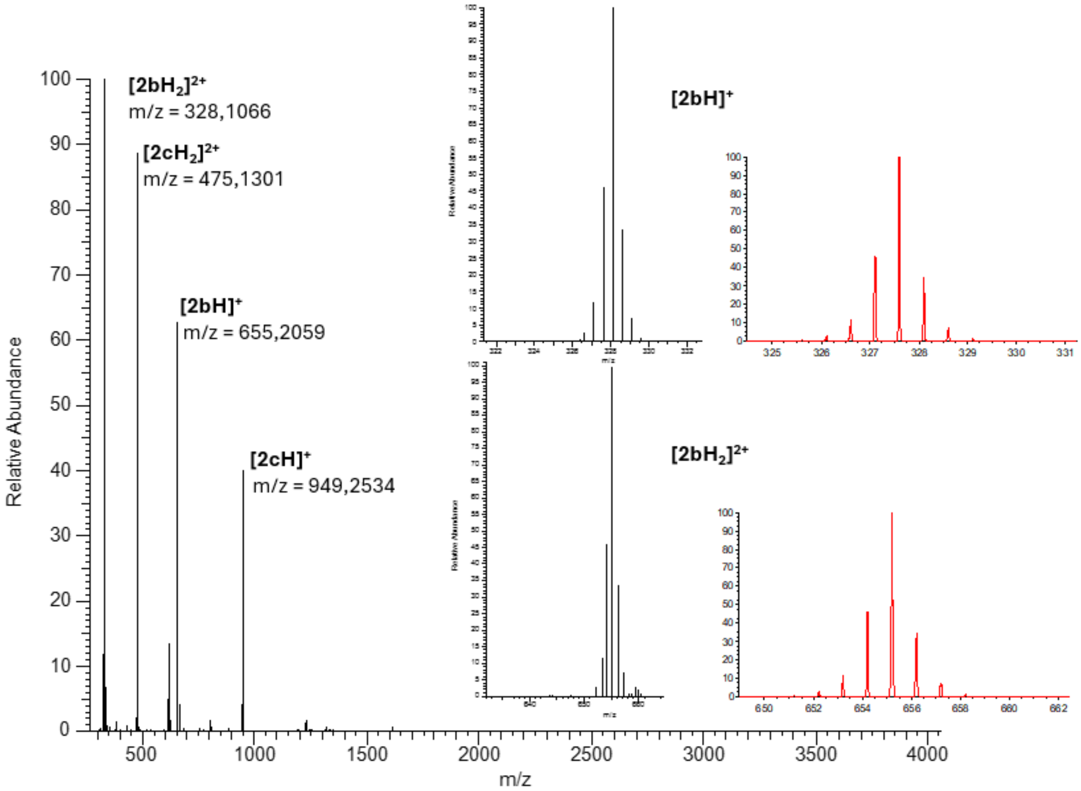

Figure S31. ESI-MS (m/z), positive mode, of **mix1**. Only the molecular ion peaks corresponding to the mono- and di-protonated metalloligands **2b** and **2c** can be detected – the corresponding experimental and calculated isotopic distributions for **2b** are shown in the insets.

.

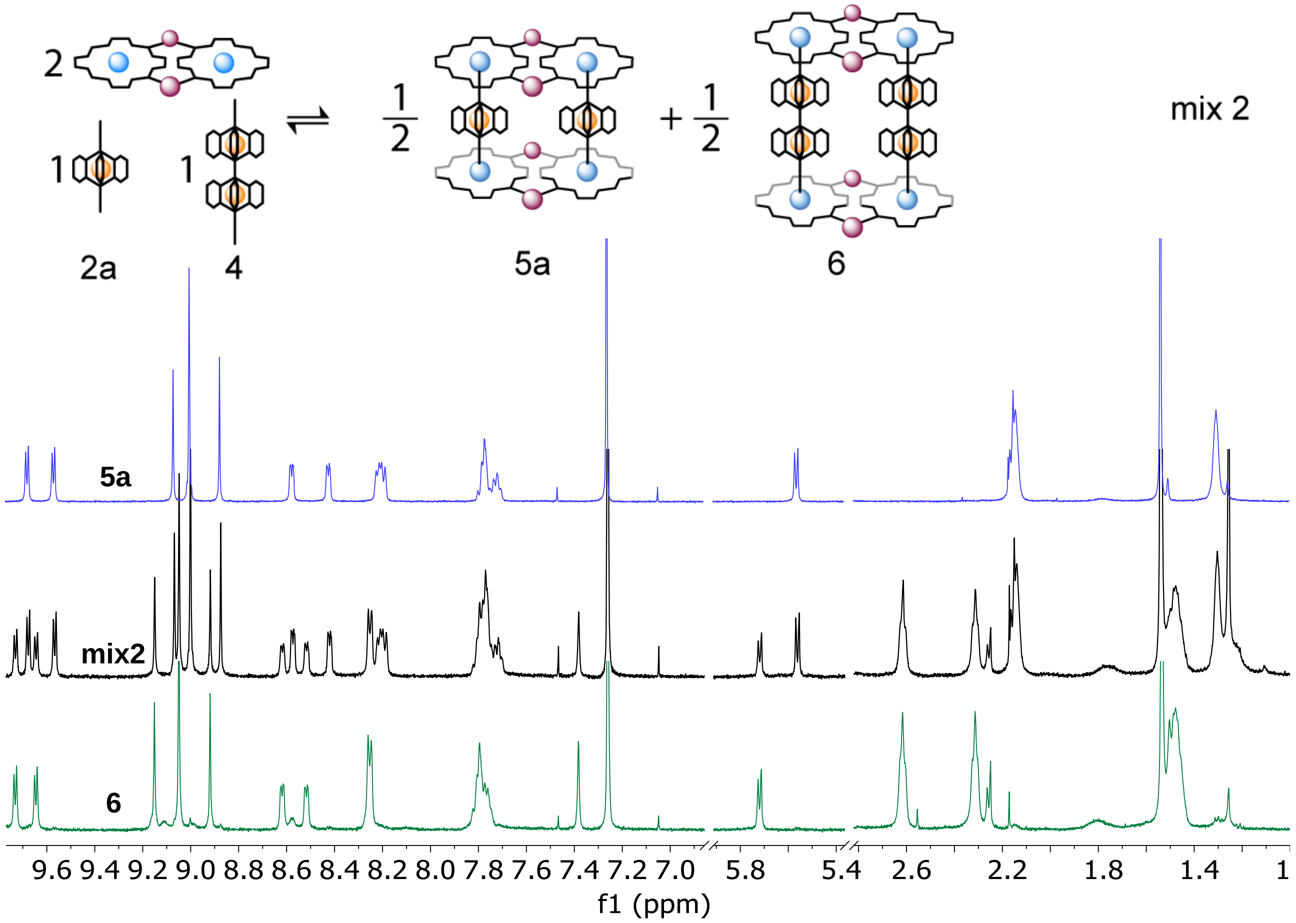

Figure S32. Top: schematic depiction of the equilibrium established by mixing **1**, **2a**, and **3** in a 2:1:1 ratio (ca. 10^{-3} M in chloroform at room temperature). Bottom: overlay of the ^{1}H NMR spectra (selected region, $CDCl_3$) of **5a** (blue); a 2:1:1 mixture of **1**, **2a**, and **3** (**mix2**, black); **6** (green).

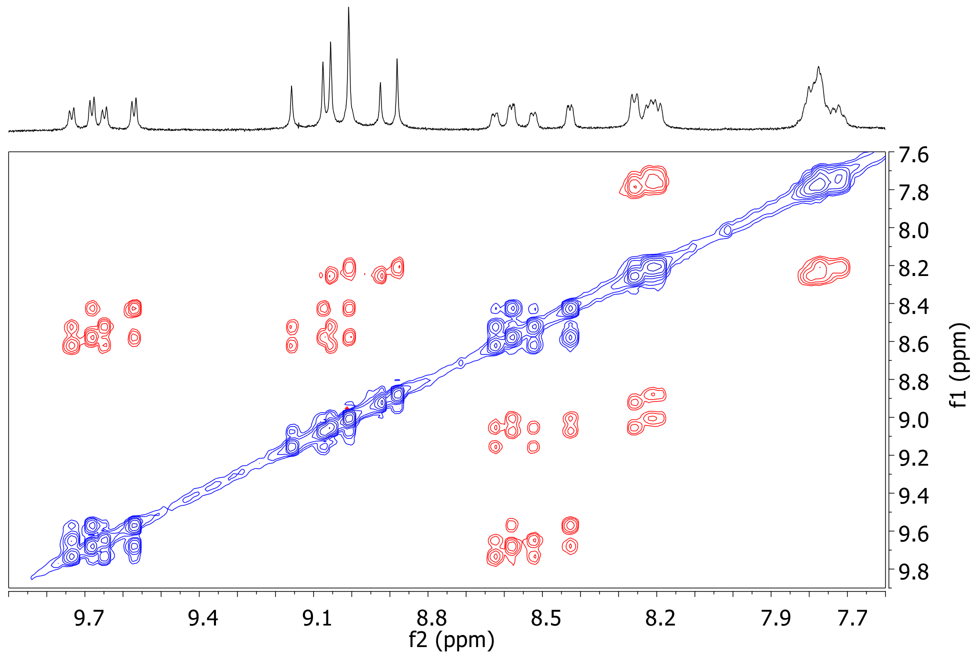

Figure S33. Aromatic region of ^{1}H-^{1}H ROESY NMR spectrum in $CDCl_3$ of **mix2.**

Emission dilution experiment.

The spectrofluorimetric dilution experiment was performed recording emission spectra of progressively more diluted chloroform solutions of **5c**, starting from a 5x10^{-5} M stock solution (λ_{exc} = 550 nm, 1 cm path length quartz cuvette). The concentration was monitored by absorption spectroscopy.

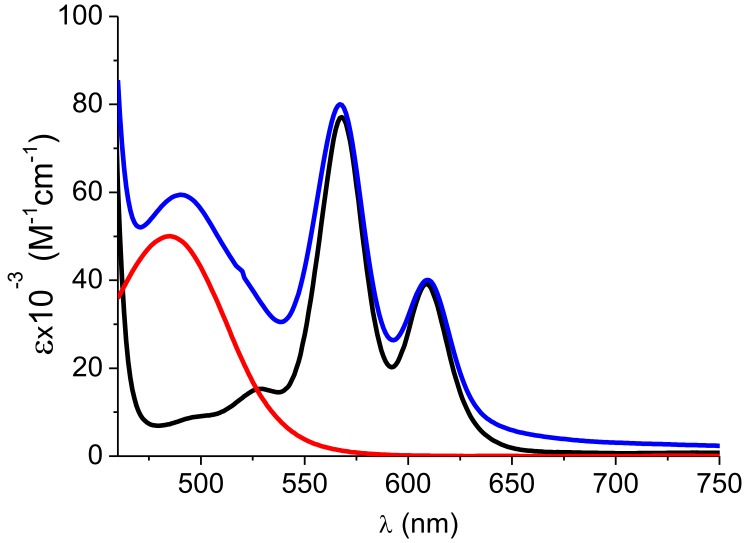

Figure S34. Absorption spectra ($CHCl_3$, visible selected region) of **1** (black line); **2c** (red line), and **5c** (blue line).

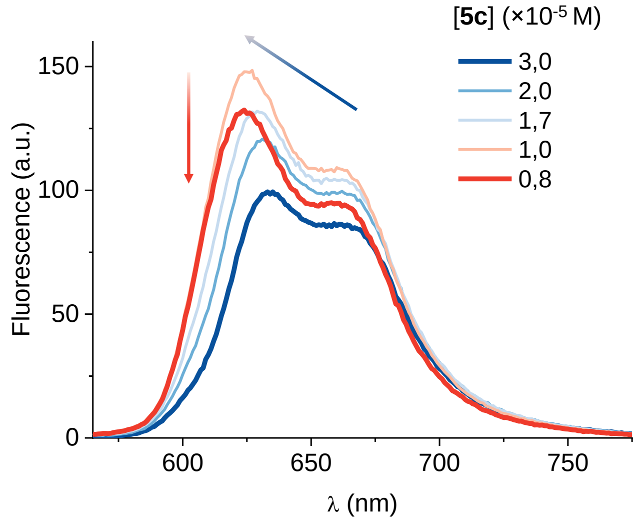

Figure S35. Emission spectra ($CHCl_3$, λ_{exc} = 550 nm) of **5c** at different concentrations.

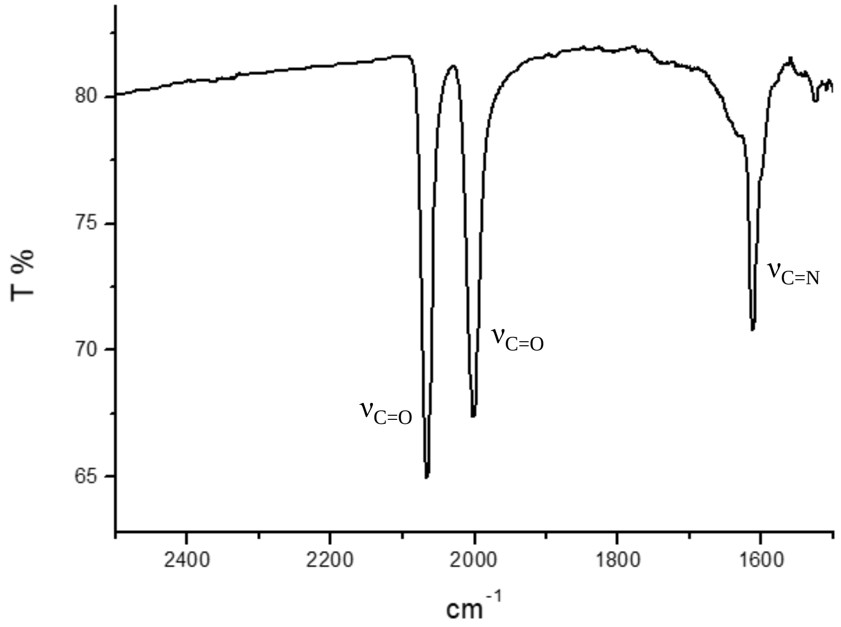

Figure S36. Selected IR stretching bands for **5c**.

X-ray Analysis.

All the assemblies consist of two metalloligands and two platforms **1**, and show an inversion center in the barycenter, therefore only half of each assembly occupy the corresponding crystallographic asymmetric units (ASU – Figure S37). An overview of the structures for the whole set, including two model compounds, is given in Figures S33-S39. The structures of the ligands **2a** and **3** in the assemblies **5a** and **6** respectively, differ from the structures of the free Fe^{II}-metalloligands previously published:[49] the rod-shaped dipyridyl connectors are slightly bent, reducing the pyridines N···N distance, and the Fe···Fe distance in **3** and **6**, Figure S37. Also, the platforms are not perfectly planar, presenting a ~4.3° angle between the 4′cisDPyP units (Figure S43). The presence of an inversion center in each assembly gives rise to a perfect parallelism between the two platforms **1**. Each Zn^{II}-porphyrin within platform **1** adopts a twisted conformation with the Zinc cation protruding by ~0.30 Å from the average macrocycle plane, in line with the coordination with the apical metalloligand pyridyl group (Table S2, and Figure S44). This conformation minimizes the steric repulsions between interleaved metalloligands and fulfill geometric constrain imposed by the axial coordination of pyridyl group to the zinc porphyrins. It cannot be excluded that part of porphyrins and ligand distortions are due to packing effects. Notable stacking effects involve phenyl rings C–H···π interactions and π···π contacts that zinc-porphyrin platforms try to maximize forming piles. Hydrophobic contacts happen through pairing of neighbor assemblies with partial lateral overlaps. In this way the metalloligands interact with porphyrins of platforms that belong to adjacent sandwiches. Solvent molecules are trapped in voids formed among packed assemblies (voids represent from 25 to 50% of cell volumes). Very often well-ordered hexane or chloroform molecules are present in between hydrophobic pockets delimited by iron-core paddles. Contacts between neighbor assemblies force ligands to adopt defined conformations. Notable discrepancies are represented by the **7** and **5a** assemblies. Structure of sandwich **7** is partially disordered: half of the ligand shows poor electron density maps, and the ligand could be modeled in two equally populated conformations.

Table S2. Selected porphyrin planes R.M.S.D. [Å], metal offset [Å] and angles between planes [°] for model compounds **2aTPP$_2$**, **2aTPP$_2$**, and the assemblies **5a-c**, **6**, and **7**.

	Zn_{porf}^{RMSD} (a)	Zn_{porf}^{Offset} (b)	$Platform_{Intra}^{Angle}$ (c)	Zn_{porf}^{Roll} (d)
**2aTPP$_2$**	0.067(13), 0.122(12)	0.3564(15)	3.383(62)	8.514(51)
**2cTPP$_2$**	0.1411(31)	0.3205(5)	16.747(20)	11.781(14)
**5a**	0.1429(73), 0.1120(76)	0.3058(11)	4.725(35)	87.289(16)
**5a'**	0.143(40), 0.167(28)	0.2916(38)	6.002(136)	81.687(59)
**5b**	0.142(22), 0.187(23)	0.2832(25)	4.910(98)	75.322(50)
**5c**	0.0978(43), 0.1821(38)	0.2797(8)	2.030(21)	89.136(7)
**6**	0.196(14), 0.148(15)	0.3222(18)	2.172(75)	89.042(28)
**7**	0.219(19), 0.082(19)	0.3194(23)	6.210(83)	88.273(39)
**8**(e)	0.135(22), 0.138(21)	0.2518(29)	16.502(124)	89.443(56)

(a)Root mean square deviation of the porphyrin core atoms from average plane. (b)Distance of Zn metal from its porphyrin macrocycle average plane. (c)Angle between the two average platform planes (each defined by Zn^{II}-porphyrins of one same platform) or between the planes of the two TPP units. (d)Angle between the average platform planes and that defined by the Zn and Fe atoms or between average TPPs plane normal vector and line through Zn and Fe. (e)Values calculated from the reported structure of the sandwich reference assembly {[*t,c,c*-$RuCl_2$(Zn·4'cisDPyP]$_2$}$_2${4'transDPyP}$_2$ (**8**, Figure S4).[15]

Table S3. Crystallographic data and refinement details for **5a**, **5a'**, **5b** and **5c**.

	**5a**·8CHCl$_3$·14C$_6$H$_{14}$ [C$_{232}$H$_{168}$B$_4$Cl$_8$Fe$_2$N$_{40}$O$_{20}$Ru$_4$ Zn$_4$·8CHCl$_3$·14C$_6$H$_{14}$]	**5a'**·10CHCl$_3$·2.5C$_6$H$_{14}$ [C$_{232}$H$_{168}$B$_4$Cl$_8$Fe$_2$N$_{40}$O$_{20}$Ru$_4$ Zn$_4$·10CHCl$_3$·2.5C$_6$H$_{14}$]	**5b**·6CHCl$_3$·2C$_6$H$_{14}$ [C$_{232}$H$_{180}$B$_4$Cl$_8$Fe$_2$N$_{40}$O$_{20}$Ru$_4$ Zn$_4$·8CHCl$_3$·14C$_6$H$_{14}$]	**5c**·17CHCl$_3$·8C$_6$H$_{14}$ [C$_{280}$H$_{180}$B$_4$Cl$_8$Fe$_2$N$_{40}$O$_{20}$Ru$_4$ Zn$_4$·17CHCl$_3$·8C$_6$H$_{14}$]
CCDC Number	2388762	2388763	2388764	2388765
Chemical Formula	C$_{324}$H$_{372}$B$_4$Cl$_{32}$Fe$_2$N$_{40}$O$_{20}$Ru$_4$ Zn$_4$	C$_{257}$H$_{213}$B$_4$Cl$_{38}$Fe$_2$N$_{40}$O$_{20}$Ru$_4$ Zn$_4$	C$_{324}$H$_{384}$B$_4$Cl$_{32}$Fe$_2$N$_{40}$O$_{20}$Ru$_4$ Zn$_4$	C$_{345}$H$_{309}$B$_4$Cl$_{59}$Fe$_2$N$_{40}$O$_{20}$Ru$_4$ Zn$_4$
Formula weight (g/mol)	7101.69	6349.46	7113.79	8247.56
Temperature (K)	100(2)	100(2)	100(2)	100(2)
Wavelength (Å)	0.700	0.700	0.700	0.700
Crystal system	Triclinic	Triclinic	Monoclinic	Triclinic
Space Group	*P* -1	*P* -1	*P* 2_1/*n*	*P* -1
Unit cell dimensions	*a* = 20.066(4) Å	*a* = 19.346(4) Å	*a* = 8.525(2) Å	*a* = 19.644(4) Å
	b = 23.667(5) Å	*b* = 20.732(4) Å	*b* = 38.882(8) Å	*b* = 22.480(5) Å
	c = 24.049(5) Å	*c* = 20.762(4) Å	*c* = 41.176(8) Å	*c* = 23.784(5) Å
	α = 65.22(3) °	α = 95.56(3)°	α = 90°	α = 103.22(3)°
	β = 85.10(3) °	β = 96.28(3)°	β = 90.85(3)°	β = 96.95(3)°
	γ = 65.02(3) °	γ = 115.68(3)°	γ = 90°	γ = 112.44(3)°
Volume (Å^3)	9341(4)	7363(3)	13647(5)	9194(4)
Z	1	1	2	1
Density (calculated) (g·cm^{-3})	1.263	1.432	1.731	1.490
Absorption coefficient (mm^{-1})	0.734	0.972	1.006	0.936
F(000)	3668	3209	7360	4190
Crystal size (mm^3)	0.03 x 0.03 x 0.01	0.03 x 0.01 x 0.01	0.02 x 0.02 x 0.01	0.05 x 0.03 x 0.01
Crystal habit	Red thin plates	Red thin needles	Red thin plates	Red thin plates
Theta range for data collection	0.92° to 24.01°	1.09° to 15.38°	0.71° to 19.28°	0.89° to 29.08°
Resolution (Å)	0.86	1.3	1.06	0.72
Index ranges	$-23 \le h \le 23$<br>$-24 \le k \le 27$<br>$0 \le l \le 27$	$-14 \le h \le 14$<br>$-15 \le k \le 15$<br>$-15 \le l \le 15$	$-8 \le h \le 8$<br>$0 \le k \le 36$<br>$0 \le l \le 38$	$-27 \le h \le 27$<br>$-31 \le k \le 30$<br>$0 \le l \le 33$
Reflections collected	86606	25024	31434	143787
Independent reflections (data with I>2σ(I))	30121 (18002)	6691 (4229)	11772 (6967)	50163 (38618)
Data	2.82 (2.81)	3.73 (3.72)	2.60 (2.39)	2.79 (2.58)

multiplicity (max resltn)				
$I/\sigma(I)$ (max resltn)	10.48 (3.27)	3.14 (2.53)	5.33 (1.96)	11.93 (5.46)
R_{merge} (max resltn)	0.1087 (0.4891)	0.1563 (0.2758)	0.1162 (0.3822)	0.0367 (0.1377)
Data completenes s (max resltn)	98.1% (97.5%)	99.9% (99.9%)	98.3% (98.6%)	97.3% (95.3%)
Refinement method	Full-matrix least-squares on F^2	Full-matrix least-squares on F^2	Full-matrix least-squares on F^2	Full-matrix least-squares on F^2
Data / restraints / parameters	30121 / 177 / 1652	6691 / 97 / 1061	11772 / 89 / 925	50163 / 259 / 2251
Goodness-of-fit on F^2	1.011	1.120	1.037	1.008
Δ/σ_{max}	0.014	0.009	0.011	0.066
Final *R* indices [$I>2\sigma(I)$]	R_1 = 0.0919, wR_2 = 0.2529	R_1 = 0.1286, wR_2 = 0.3036	R_1 = 0.1202, wR_2 = 0.2926	R_1 = 0.0845, wR_2 = 0.2474
R indices (all data)	R_1 = 0.1436, wR_2 = 0.2993	R_1 = 0.1791, wR_2 = 0.3538	R_1 = 0.1857, wR_2 = 0.3393	R_1 = 0.1016, wR_2 = 0.2644
Largest diff. peak and hole ($eÅ^{-3}$)	0.951 and −0.948	0.874 and −0.457	1.224 and −0.712	2.281 and −1.978
R.M.S. deviation from mean ($eÅ^{-3}$)	0.121	0.120	0.143	0.139
Squeezed electrons (e/cell)	616	42	193	431
Squeezed Voids Volume ($Å^3$/cell) (%)	2429 (26.0%)	296 (4.0%)	1081 (7.9%)	1428 (15.5%)
Total Solvent Voids Volume ($Å^3$/cell) (%)	4798 (51.4%)	2879 (39.1%)	3453 (25.3%)	3993 (43.4%)

$R_1 = \Sigma \,||Fo|-|Fc|| \,/\, \Sigma\, |Fo|$; $wR_2 = \{\Sigma\, [w(Fo^2 - Fc^2)^2] \,/\, \Sigma\, [w(Fo^2)^2]\}^{½}$

Table S3 (cont.). Crystallographic data and refinement details for **6** and **7**.

	**6**·$10CHCl_3·19C_6H_{14}$ [$C_{280}H_{224}B_8Cl_8Fe_4N_{52}O_{32}Ru_4$ $Zn_4·10CHCl_3·19C_6H_{14}$]	**7**·$30CHCl_3·6C_6H_{14}$ [$C_{292}H_{232}B_8Cl_8Fe_4N_{52}O_{32}Ru_4$ $Zn_4·30CHCl_3·6C_6H_{14}$]
CCDC Number	2388766	2388767
Chemical Formula	$C_{404}H_{500}B_8Cl_{38}Fe_4N_{52}O_{32}Ru_4 Zn_4$	$C_{358}H_{346}B_8Cl_{98}Fe_4N_{52}O_{32}Ru_4 Zn_4$
Formula weight (g/mol)	8919.27	10338.59
Temperature (K)	100(2)	100(2)
Wavelength (Å)	0.700	0.700
Crystal system	Triclinic	Triclinic
Space Group	*P* -1	*P* -1
Unit cell dimensions	*a* = 21.169(4) Å	*a* = 18.739(4) Å
	b = 24.320(5) Å	*b* = 22.432(4) Å
	c = 25.085(5) Å	*c* = 34.773(7) Å
	α = 71.92(3)°	α = 93.97(3)°
	β = 78.09(3)°	β = 98.48(3)°
	γ = 78.51(3)°	γ = 113.24(3)°
Volume (Å^3)	11885(5)	13152(5)
Z	1	1
Density (calculated) (g·cm^{-3})	1.246	1.305
Absorption coefficient (mm^{-1})	0.679	0.901
F(000)	4630	5220
Crystal size (mm^3)	0.03 x 0.03 x 0.01	0.03 x 0.03 x 0.01
Crystal habit	Red thin plates	Red thin plates
Theta range for data collection	0.85° to 22.36°	1.22° to 20.07°
Resolution (Å)	0.92	1.02
Index ranges	$-23 \le h \le 22$ $-26 \le k \le 26$ $-26 \le l \le 27$	$-18 \le h \le 18$ $-21 \le k \le 21$ $-34 \le l \le 34$
Reflections collected	91167	93481
Independent reflections (data with I>2σ(I))	30935 (15321)	25660 (12973)
Data multiplicity (max resltn)	2.86 (2.12)	3.61 (3.57)
I/σ(I) (max resltn)	6.97 (1.43)	4.58 (1.40)
R_{merge} (max resltn)	0.0857 (0.5018)	0.1173 (0.5347)
Data completeness (max resltn)	96.9% (92.9%)	99.0% (98.9%)
Refinement method	Full-matrix least-squares on F^2	Full-matrix least-squares on F^2
Data / restraints / parameters	30935 / 197 / 2004	25660 / 515 / 2032
Goodness-of-fit on F^2	1.043	0.944
Δ/σ_{max}	0.024	0.027
Final *R* indices [I>2σ(I)]	R_1 = 0.1190, wR_2 = 0.2867	R_1 = 0.1309, wR_2 = 0.2796
R indices (all data)	R_1 = 0.1897, wR_2 = 0.3302	R_1 = 0.1973, wR_2 = 0.3253
Largest diff. peak and hole (eÅ^{-3})	1.060 and −0.746	1.118 and −0.851
R.M.S. deviation from mean (eÅ^{-3})	0.110	0.105
Squeezed electrons (e/cell)	866	1160
Squeezed Voids Volume (Å^3/cell) (%)	3229 (27.2%)	3893 (29.6%)
Total Solvent Voids Volume (Å^3/cell) (%)	6237 (52.5%)	3903 (29.7%)

$R_1 = \Sigma ||F_o|-|F_c|| / \Sigma |F_o|$; $wR_2 = \{\Sigma [w(F_o^2 - F_c^2)^2] / \Sigma [w(F_o^2)^2]\}^{1/2}$

Table S3 (cont.). Crystallographic data and refinement details for **2aTPP$_2$** and **2cTPP$_2$**.

	**2aTPP$_2$**·6C_6H_{14} [$C_{116}H_{88}B_2FeN_{16}O_6Zn_2$·6$C_6H_{14}$]	**2cTPP$_2$**·3$CHCl_3$ [$C_{140}H_{94}B_2FeN_{16}O_6Zn_2$·3$CHCl_3$]
CCDC Number	2388760	2388761
Chemical Formula	$C_{152}H_{172}B_2FeN_{16}O_6Zn_2$	$C_{143}H_{97}B_2Cl_9FeN_{16}O_6Zn_2$
Formula weight (g/mol)	2527.26	2662.62
Temperature (K)	100(2)	100(2)
Wavelength (Å)	0.71073	0.700
Crystal system	Triclinic	Monoclinic
Space Group	*P* -1	*I* 2/*a*
Unit cell dimensions	*a* = 14.058(6) Å	*a* = 19.074(4) Å
	b = 20.156(10) Å	*b* = 24.641(5) Å
	c = 24.184(5) Å	*c* = 26.708(5) Å
	α = 107.66(3)°	α = 90°
	β = 99.59(3)°	β = 95.63(3)°
	γ = 103.18(3)°	γ = 90°
Volume (Å^3)	6149(4)	12493(4)
Z	2	4
Density (calculated) (g·cm^{-3})	1.365	1.416
Absorption coefficient (mm^{-1})	0.572	0.719
F(000)	2680	5456
Crystal size (mm^3)	0.27 x 0.22 x 0.09	0.08 x 0.02 x 0.02
Crystal habit	Purple plates	Red thin rods
Theta range for data collection	1.11° to 20.00°	1.11° to 30.00°
Resolution (Å)	1.04	0.70
Index ranges	$-13 \le h \le 13$<br>$-19 \le k \le 19$<br>$-23 \le l \le 23$	$-25 \le h \le 27$<br>$-33 \le k \le 33$<br>$-38 \le l \le 38$
Reflections collected	39572	65031
Independent reflections (data with I>2σ(I))	11394 (6977)	18736 (12704)
Data multiplicity (max resltn)	3.45 (2.54)	3.37 (2.96)
I/σ(I) (max resltn)	6.14 (2.17)	11.13 (3.98)
R_{merge} (max resltn)	0.1197 (0.3276)	0.0785 (0.2631)
Data completeness (max resltn)	99.2% (97.8%)	97.9% (93.8%)
Refinement method	Full-matrix least-squares on F^2	Full-matrix least-squares on F^2
Data / restraints / parameters	11394 / 2113 / 1397	18736 / 12 / 805
Goodness-of-fit on F^2	1.058	1.022
Δ/σ_{max}	0.000	0.001
Final *R* indices [I>2σ(I)]	R_1 = 0.1036, wR_2 = 0.2681	R_1 = 0.0686, wR_2 = 0.1802
R indices (all data)	R_1 = 0.1601, wR_2 = 0.3195	R_1 = 0.1060, wR_2 = 0.2049
Largest diff. peak and hole (eÅ^{-3})	0.539 and -0.603	1.142 and -1.681
R.M.S. deviation from mean (eÅ^{-3})	0.131	0.112
Squeezed electrons (e/cell)	618	N.A.
Squeezed Voids Volume (Å^3/cell) (%)	1762 (28.7%)	N.A.
Total Solvent Voids Volume (Å^3/cell) (%)	1762 (28.7%)	2120 (17.0%)

$$R_1 = \Sigma \, ||Fo|-|Fc|| \, / \, \Sigma \, |Fo|; \; wR_2 = \{\Sigma \, [w(Fo^2 - Fc^2)^2] \, / \, \Sigma \, [w(Fo^2)^2]\}^{½}$$

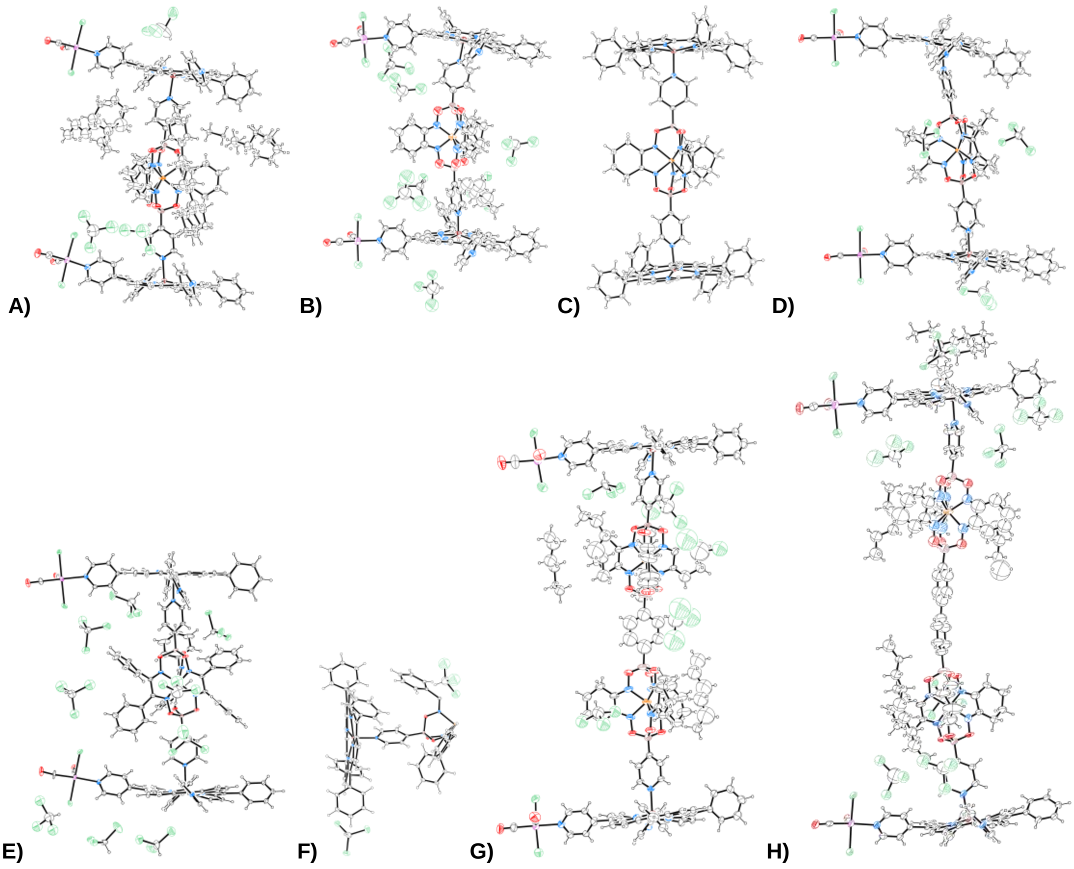

Figure S37. Ellipsoids representation of ASU contents (50% probability) for: A) **5a**, B) **5a′**, C) **2aTPP$_2$**, D) **5b**, E) **5c** F) **2cTPP$_2$**, G) **6** and H) **7**. Disordered conformations omitted for clarity.

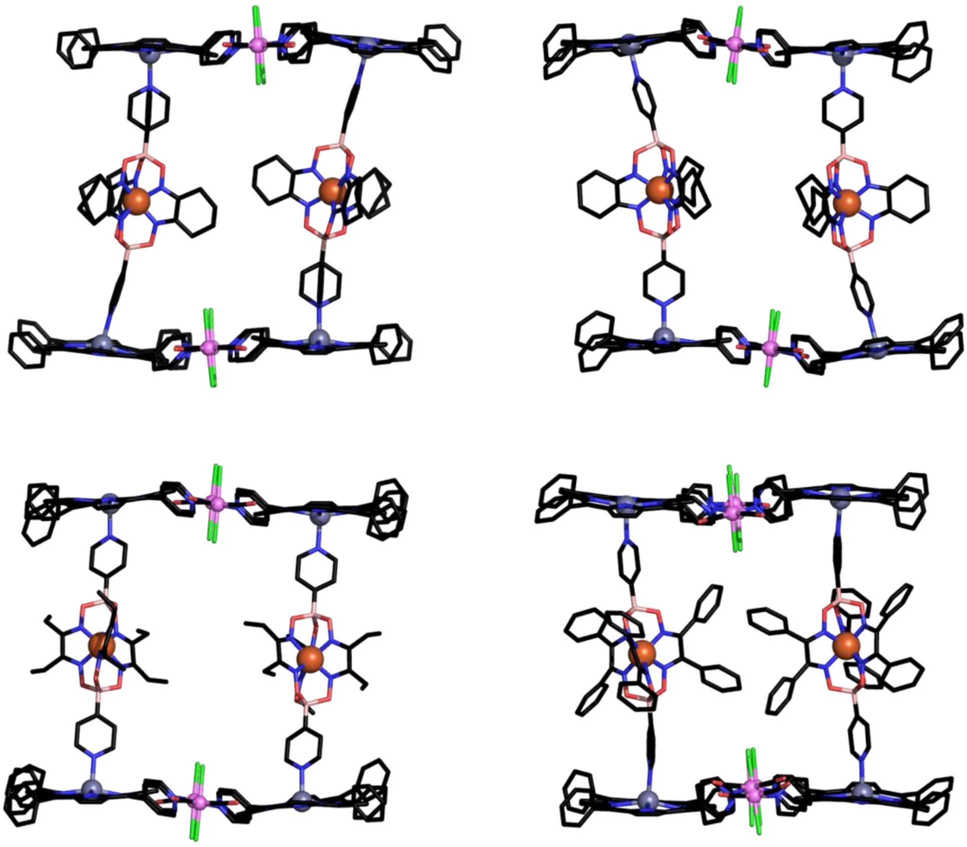

Figure S38. Molecular structures of assemblies **5a**, **5a′** (top, left and right, respectively), **5b** and **5c** (bottom, left and right, respectively); solvent molecules and hydrogen atoms are omitted for clarity. Color coding: black sticks for C, blue for N, red for O, pink for B, violet for Ru, orange for Fe, purple for Zn, green for Cl.

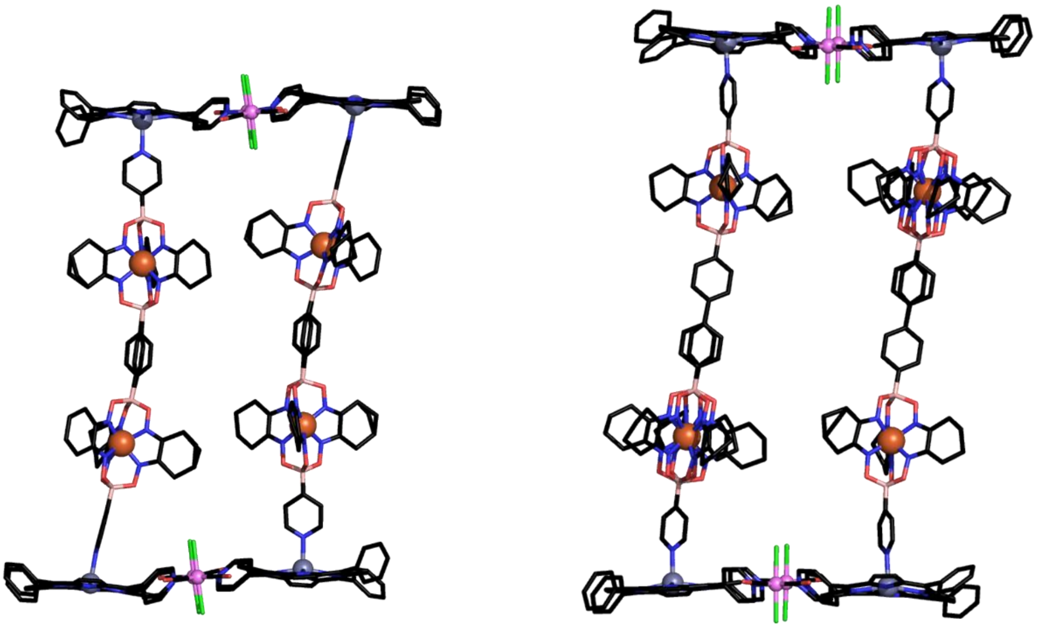

Figure S39. Molecular structures of assemblies **6** and **7** (left and right, respectively); solvent molecules and hydrogen atoms are omitted for clarity. Color coding: black sticks for C, blue for N, red for O, pink for B, violet for Ru, orange for Fe, purple for Zn, green for Cl.

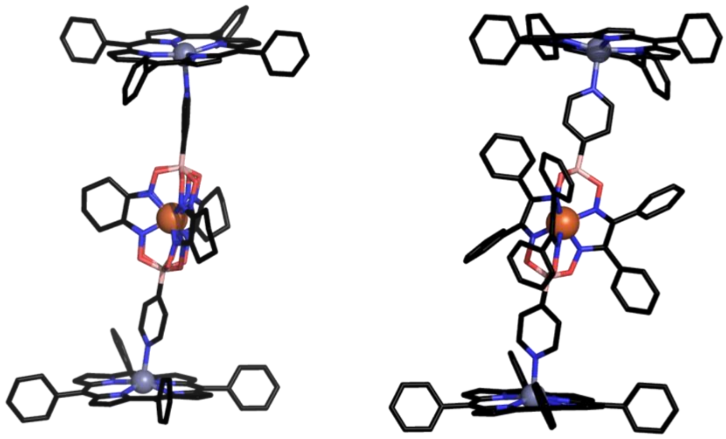

Figure S40. Molecular structures of model compounds **2aTPP$_2$** and **2cTPP$_2$** (left and right, respectively); solvent molecules and hydrogen atoms are omitted for clarity. Color coding: black sticks for C, blue for N, red for O, pink for B, orange for Fe, purple for Zn.

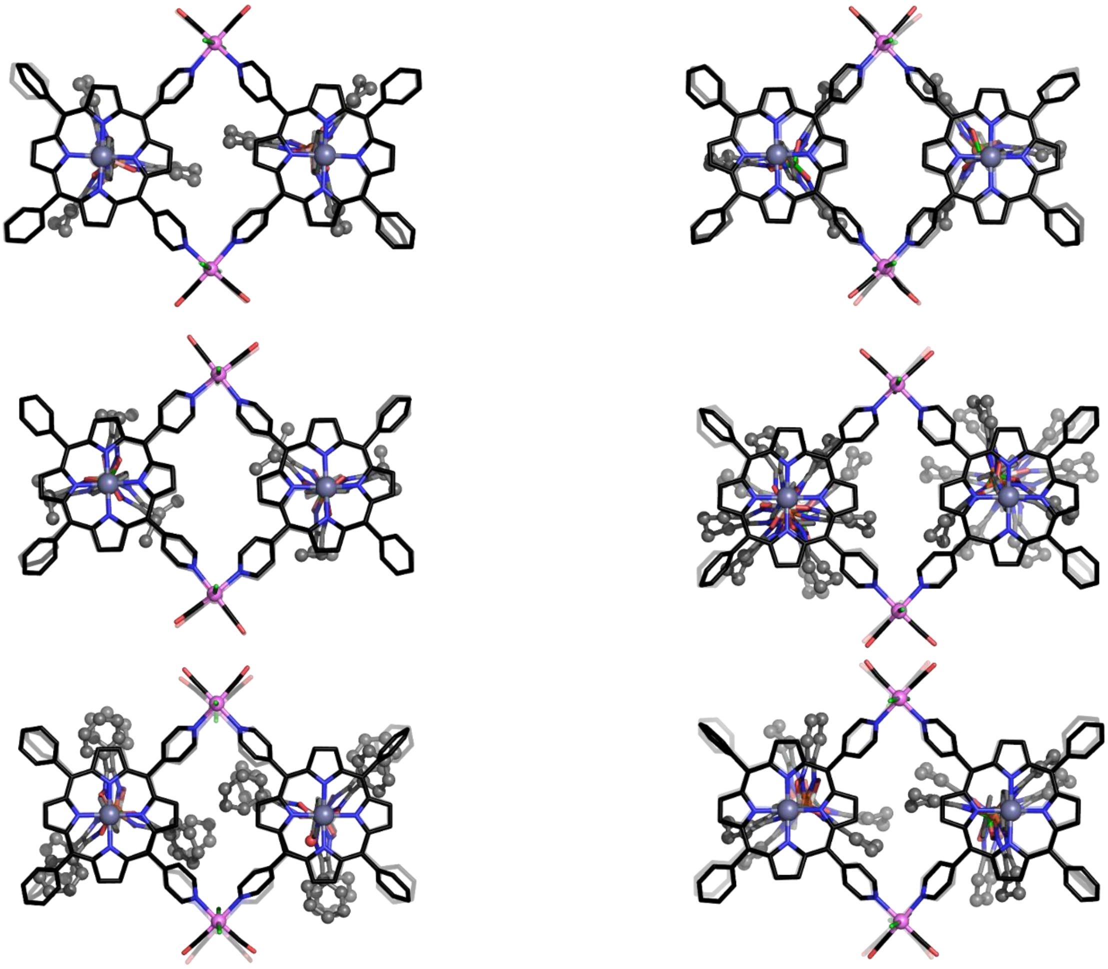

Figure S41. Top views of assemblies **5-7** (Fe^{II}-metalloligands carbon skeleton depicted in grey).

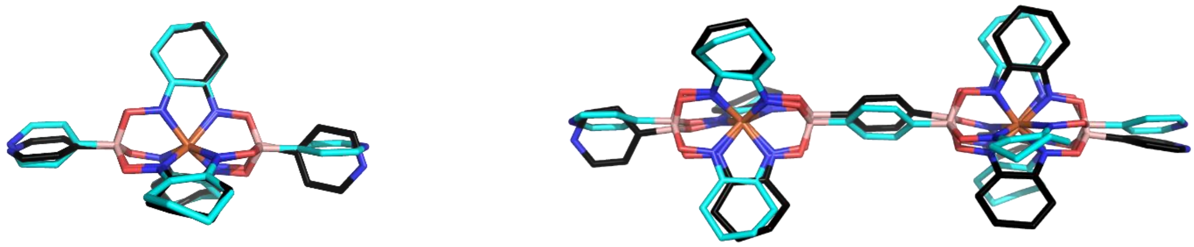

Figure S42. Left: overlap between structures of **2a** as free Fe^{II}-metalloligand[45] or as connector in **5a′** (carbon skeleton in black or light blue sticks, respectively). Right: Overlap between structures of **3** as free Fe^{II}-metalloligand[45] or as connector in **6** (carbon skeleton in black or light blue sticks, respectively).

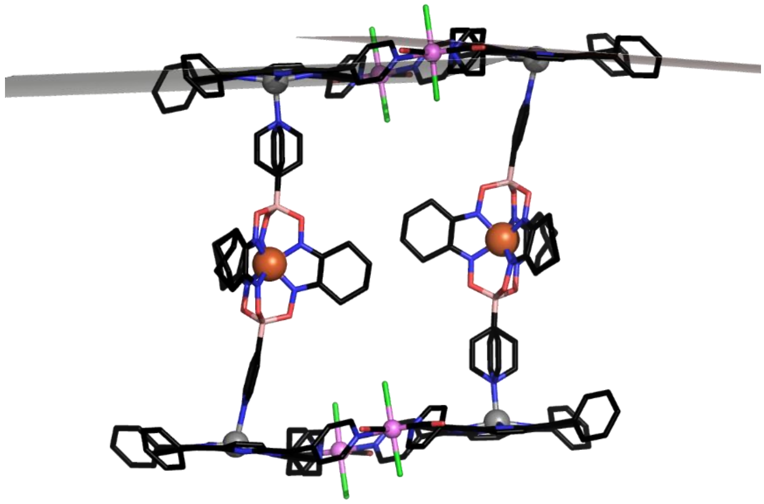

Figure S43. Molecular structure of **5a** showing the angle between the average planes containing Zn^{II}-porphyrin units of the same Ru^{II}-metallacycle **1** (4.72(4)°). The zinc cation displacement can be also appreciated (0.306(1) Å).

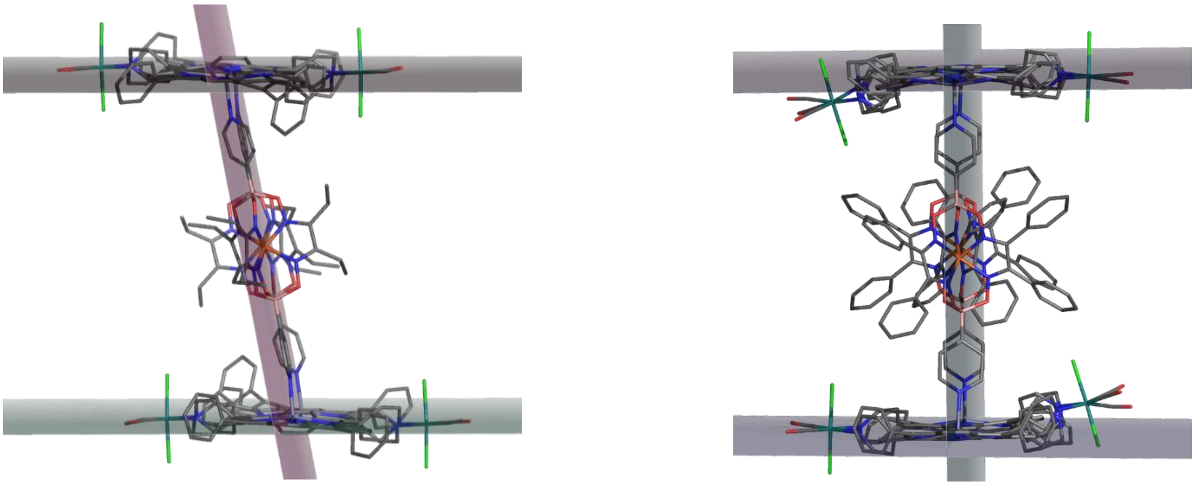

Figure S44. Qualitative relative disposition of the average planes containing the two facing platforms and the connecting Fe^{II}-metalloligands for **5b** (left) and **5c** (right). The largest deviation from orthogonality is found for **5b**.

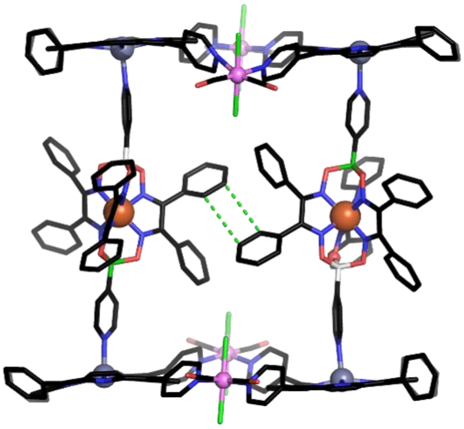

Figure S45. Molecular structure of **5c**, showing the favorable staking interactions observed for the inner phenyl substituents of two distinct Fe^{II}-metalloligands **2c** (shortest C···C distance is of 3.68 Å).
